# Supplementary material for: Next generation synthetic memory via intercepting recombinase function
Source: Nat Commun. 2023 Aug 29;14:5255. doi: 10.1038/s41467-023-41043-w (PMC10465543; doi:10.1038/s41467-023-41043-w)
Supplement: Supplementary file 1 — Supplementary Information [file 41467_2023_41043_MOESM1_ESM.pdf]

Supplementary Information

Next generation synthetic memory *via* intercepting recombinase function

Andrew E. Short[1]*, Dowan Kim[1]*, Prasaad T. Milner[1], and Corey J. Wilson[1†]

[1]Georgia Institute of Technology, School of Chemical & Biomolecular Engineering

*authors contributed equally

[†]To whom correspondence should be addressed: Corey J. Wilson, Georgia Institute of Technology, School of Chemical & Biomolecular Engineering, 311 Ferst Drive, Atlanta, GA 30332-0100. E-Mail: corey.wilson@chbe.gatech.edu

Supplementary Note 1: Intelligent biotic system – definition.

Supplementary Note 2: Defining moderate conditions.

Supplementary Note 3: First design rule for the interception (deletion) memory circuit.

Supplementary Note 4: Second design rule for the interception (deletion) memory circuit.

Supplementary Figure 1: Illustrations and iconography for deletion and inversion synthetic memory.

Supplementary Figure 2: Definition and analysis of recombinase attachment half-site omission for A118, TP901, Int2, and Int3.

Supplementary Figure 3: Definition and analysis of recombinase attachment half-site omission for Int5, Int8, Int12, and Bxb1.

Supplementary Figure 4: Qualitative genotype of 8 recombinases paired with cognate interception deletion circuits.

Supplementary Figure 5: Transcriptional repressor performance abstraction and sequence similarity between each operator substituted *attP* site relative to wildtype *attP*.

Supplementary Figure 6: Transcriptional repressor (E^+ variants) performance compared to repressor interception.

Supplementary Figure 7: Transcriptional repressor (R^+ variants) performance compared to repressor interception.

Supplementary Figure 8: Transcriptional repressor (F^+ variants) performance compared to repressor interception.

Supplementary Figure 9: Transcriptional repressor (G^+ variants) performance compared to repressor interception.

Supplementary Figure 10: Transcriptional repressor (I^+ variants) performance compared to repressor interception.

Supplementary Figure 11: Detailed sequences for substituted *attP* sites.

Supplementary Figure 12: Flow cytometry of select interception circuits.

Supplementary Figure 13: Ribosome binding site (RBS) tuning of A118 for three R^+ ADR.

Supplementary Figure 14: Transcriptional anti-repressor (I^A variants) performance compared to anti-repressor interception.

Supplementary Figure 15: Transcriptional anti-repressor (R^A variants) performance compared to anti-repressor interception.

Supplementary Figure 16: Transcriptional anti-repressor (F^A variants) performance compared to anti-repressor interception.

Supplementary Figure 17: Transcriptional anti-repressor (S^A variants) performance compared to anti-repressor interception.

Supplementary Figure 18: Transcriptional anti-repressor (P^A variants) performance compared to anti-repressor interception.

Supplementary Figure 19: Ribosome binding site (RBS) tuning of A118 for three $R^{A(1)}$ ADR.

Supplementary Figure 20: Nested BUFFER logic paired with interception memory.

Supplementary Figure 21: Nested NOT logic paired with interception memory.

Supplementary Figure 22: Interception synthetic memory with nested AND / NOR Boolean Logic.

Supplementary Figure 23: Additional operator positions, interception *via* A118, TP901, Int3, Int5, and Int12.

Supplementary Figure 24: Interception performance with different central dinucleotides.

Supplementary Figure 25: Double-layer deletion circuit with two orthogonal attachment sites.

Supplementary Figure 26: Flow cytometry of a two-output circuit.

Supplementary Figure 27: Gating method for flow cytometry.

Supplementary Figure 28: Comparing type-I memory and type-II memory kinetics with constant INPUT.

Supplementary Figure 29: Comparing type-I memory and type-II memory kinetics with transient INPUT.

Supplementary Figure 30: Comparing type-I memory and type-II memory kinetics.

Supplementary Figure 31: Interception *via* TetR.

Supplementary Figure 32: Relevant plasmid maps used in this study.

Supplementary Table 1: Maintenance of interception of Loss of Function circuits over three days.

Supplementary Note 1: Intelligent biotic system – definition. We define an intelligent biotic system as one or more chassis cells capable of (i) decision-making, (ii) coupled memory development, (iii) and – for advanced systems – communication between chassis cells and/or the host.

Supplementary Note 2: Defining moderate conditions. At the outset, we were interested in evaluating recombinase functions under moderate conditions – opposed to designing optimized circuits – accordingly the promoter strength, RBS strength, and plasmid copy numbers were fixed. Namely, all recombinase plasmids (pSK001 – pSK012) have a medium copy number p15 origin of replication, and the promoter and RBS upstream of each recombinase is fixed (see **Supplementary Figure 32**).

Supplementary Note 3: First design rule for the interception (deletion) memory circuit. Given, that the A118 system displayed a high tolerance to half-site omission (**Fig 2a**), we conducted a coarse-grained scan of operator substitution across the *attP* site, without modification to the central conserved dinucleotide AA. Noting that recombinases require identical central conserved sites in both the *attB* and *attP* for recombination, thus varying the central conserved region of the *attP* attachment site would generate a non-functional set. The justification for focusing on the *attP* attachment site was two-fold: (i) this design maintained deconvoluted regulation of the promoter, and (ii) this design facilitated independent recombinase interception. In the second criteria placing an operator in proximity to the promoter could result in regulation of the output gene *via* blocking the RNA polymerase. Accordingly, in this iteration of the synthetic memory circuit we avoided modification of the attachment site proximal to the promoter (*i.e.*, *attB*). In the scanning experiment given in **Fig. 2l**, the O^{ttg} operator was placed at 7 disparate positions (P-24, P-18, P-15, P-14, P+1, P+3, and P+4 – *i.e.*, without changing the central dinucleotide), and paired with the E^{+}_{HQN} transcription factor, with constitutive A118 recombinase expression. The downstream numbering convention is based on the position of the first base pair of the operator, relative to the first base pair of the central motif. Once the complete operator is beyond the central motif the numbering is reset to 1 relative to the last base pair of the conserved region.

Supplementary Note 4: Second design rule for the interception (deletion) memory circuit. The (i) performance metrics for each TF and (ii) qualitative catalytic efficiency of modified attachment sites dictate the performance of a given synthetic (interception) memory circuit. However, (iii) the increased catalytic efficiency of a given *attP* site can be mitigated with sufficient binding function of the TF. The (iii) criteria is predicated on the observations made for the GKR | *attP*-O^{gac} set in **Fig. 3b-f**. (also see **Supplementary Figs. 5-12**). Namely, while the memory circuits that utilize the R^{+}_{GKR}, F^{+}_{GKR}, G^{+}_{GKR}, and I^{+}_{GKR} transcription factors all exhibit low interception performance, the E^{+}_{GKR} iteration displays relatively strong interception performance. Correspondingly, the E^{+}_{GKR} has the best performance metrics in the GKR class of engineered TFs.

Supplementary Fig. 1

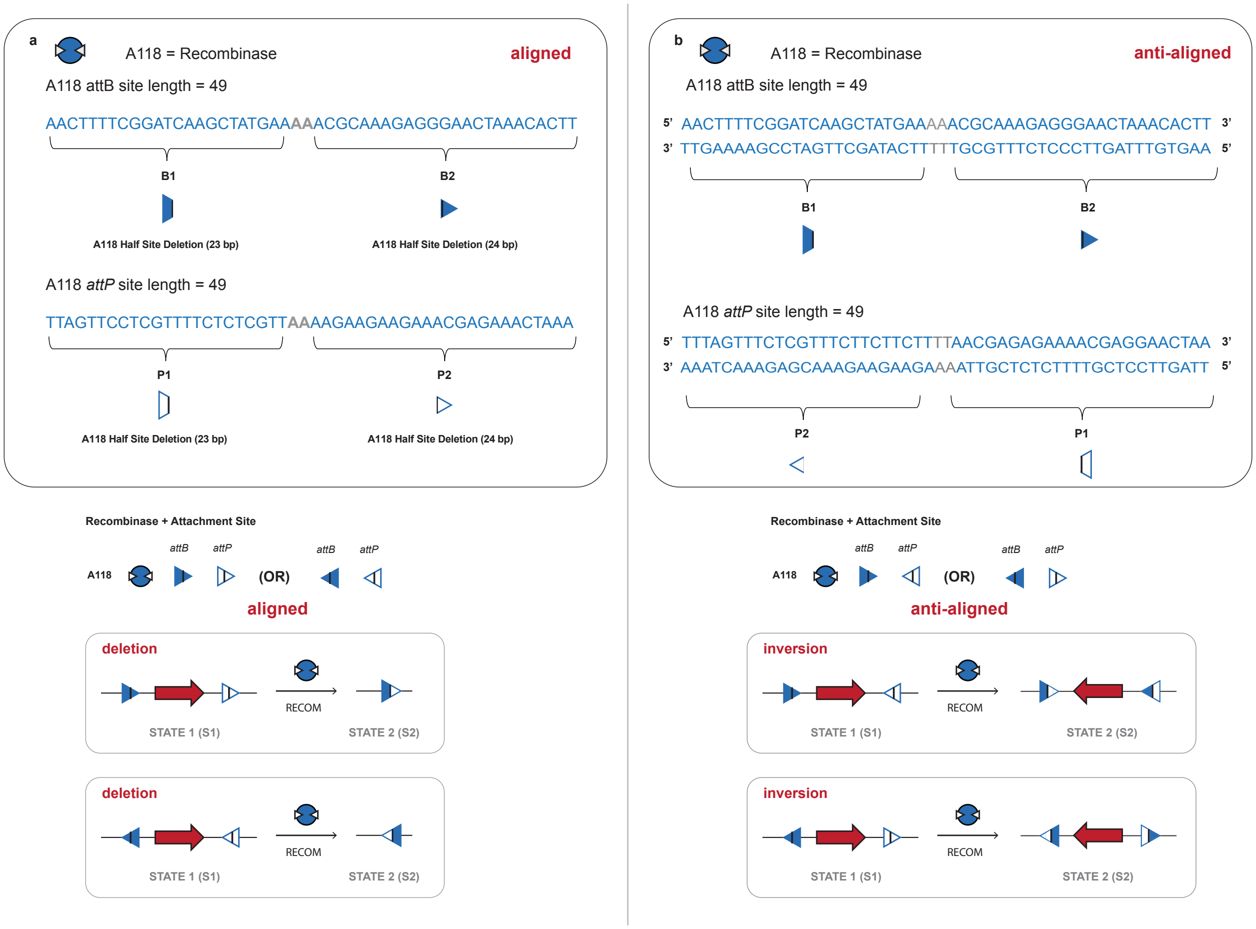

Supplementary Figure 1: Illustrations and iconography for deletion and inversion synthetic memory. a (left) A granular description of the set if A118 recombinase attachment sites in the aligned (deletion) configuration. (right) The iconography for the two aligned orientations that result in deletion. **b** (top) A detailed description of anti-aligned (inversion) attachment sites for the A118 recombinase, and below is the iconography for the two anti-aligned orientations that result in inversion. Note: The icon for the recombinase is given as a monomer.

Supplementary Fig. 2

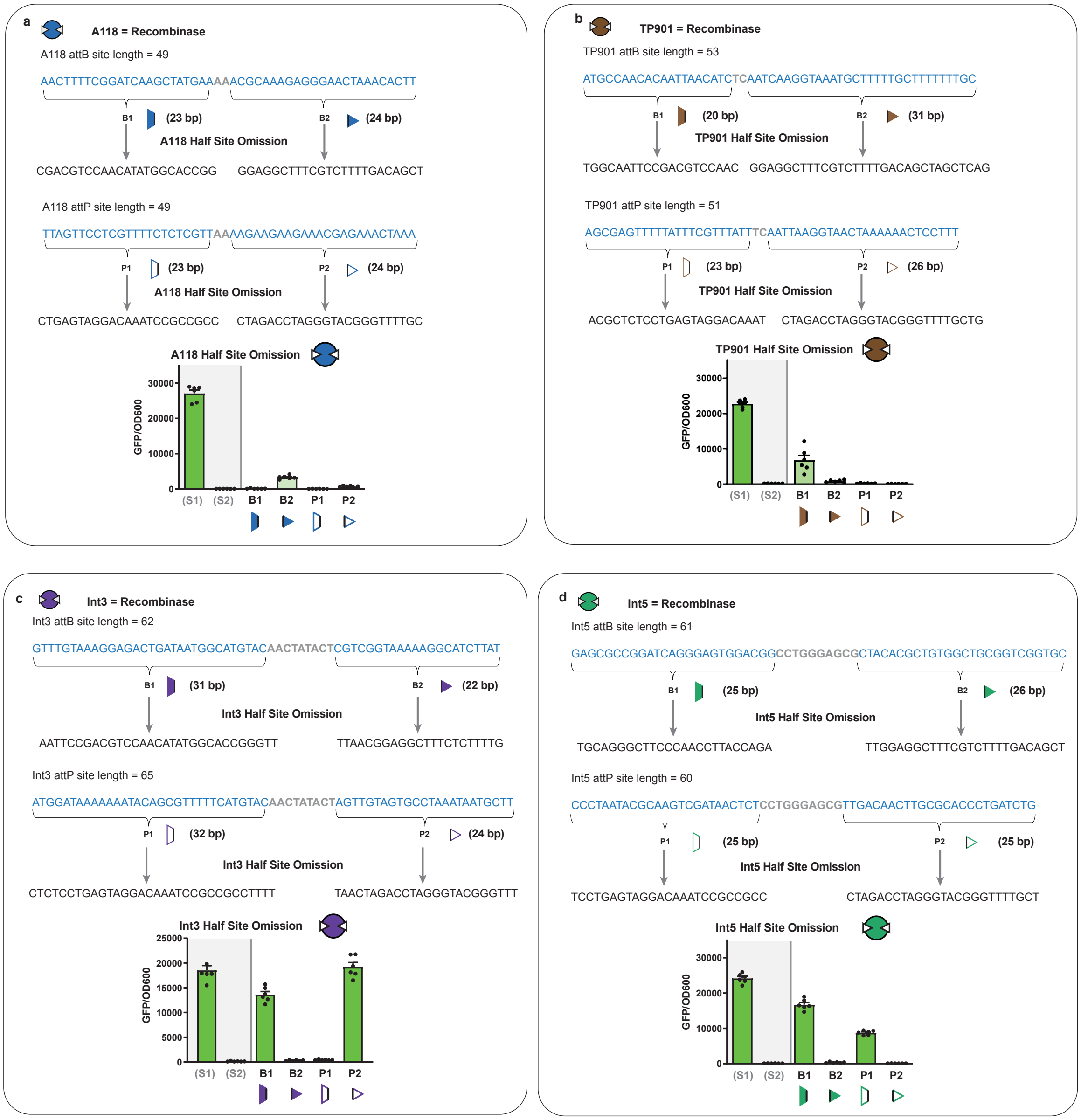

Supplementary Figure 2: Definition and analysis of recombinase attachment half-site omission for A118, TP901, Int2, and Int3. Each recombinase recognizes and recombines two attachment sites, *attB* and *attP*. Each attachment site contains a central conserved region, shown in bold grey, such that *attB* and *attP* must be identical when aligned for deletion or complementary when aligned for inversion[1] (also see **Supplementary Figure 1**). Attachment site sequences are shown in blue, and central conserved regions are shown in bold grey. **a** Symbols corresponding to recombinase half-attachment-site sequence omissions for A118. For example, A118 B1 corresponds to a truncated attachment site lacking the indicated sequence, *i.e.* **AA**ACGCAAAGAGGGAACTAAACACTT. In other words, the truncated triangle symbol refers schematically to the segment of the attachment site sequence that has been omitted. The DNA sequence on the original reporter construct (pSK001) upstream of *attB* is now present in place of the half-site B1. At right, data corresponding to the half-site omission experiment described in **Fig. 2** is shown. **b-d** The attachment site sequences and corresponding half-site sequence omissions are shown with the relevant assay data for the recombinases A118, TP901, Int2, Int3, and Int5. Source Data are provided as a Source Data file. Data in **a-d** represent the average of $n = 6$ biological replicates. Error bars correspond to the SEM of these measurements.

Supplementary Fig. 3

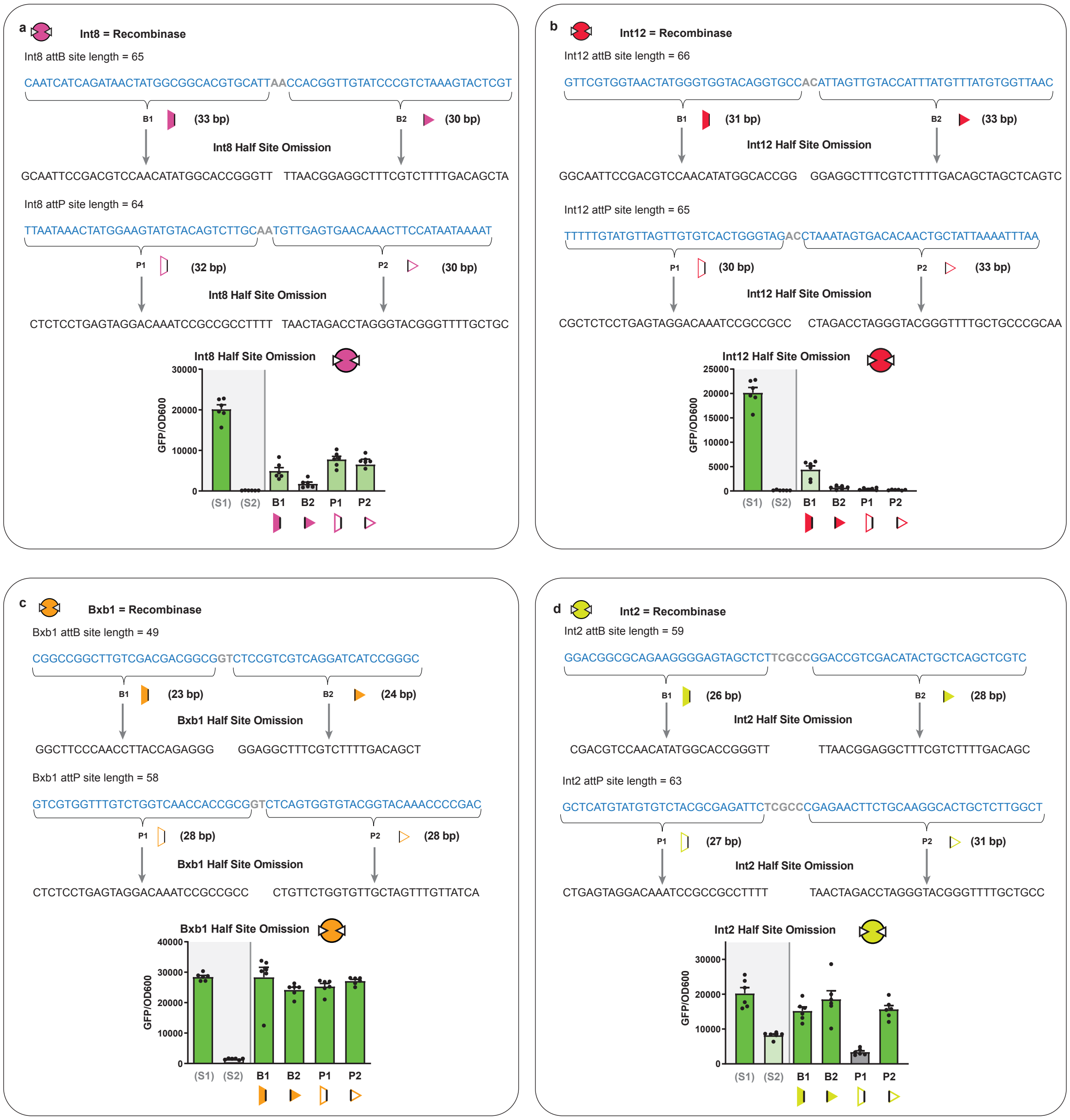

Supplementary Figure 3: Definition and analysis of recombinase attachment half-site omission for Int5, Int8, Int12, and Bxb1. Each recombinase recognizes and recombines two attachment sites, *attB* and *attP*. Each attachment site contains a central conserved region, shown in bold grey, such that *attB* and *attP* must be identical when aligned for deletion or complementary when aligned for inversion[1] (also see **Supplementary Figure 1**). Attachment site sequences are shown in blue, and central conserved regions are shown in bold grey. **a-d** The attachment site sequences and corresponding half-site sequence omissions are shown with the relevant assay data for the recombinases Int5, Int8, Int12, and Bxb1. Source data are provided as a Source Data file. Data in **a-d** represent the average of n = 6 biological replicates. Error bars correspond to the SEM of these measurements.

Supplementary Fig. 4

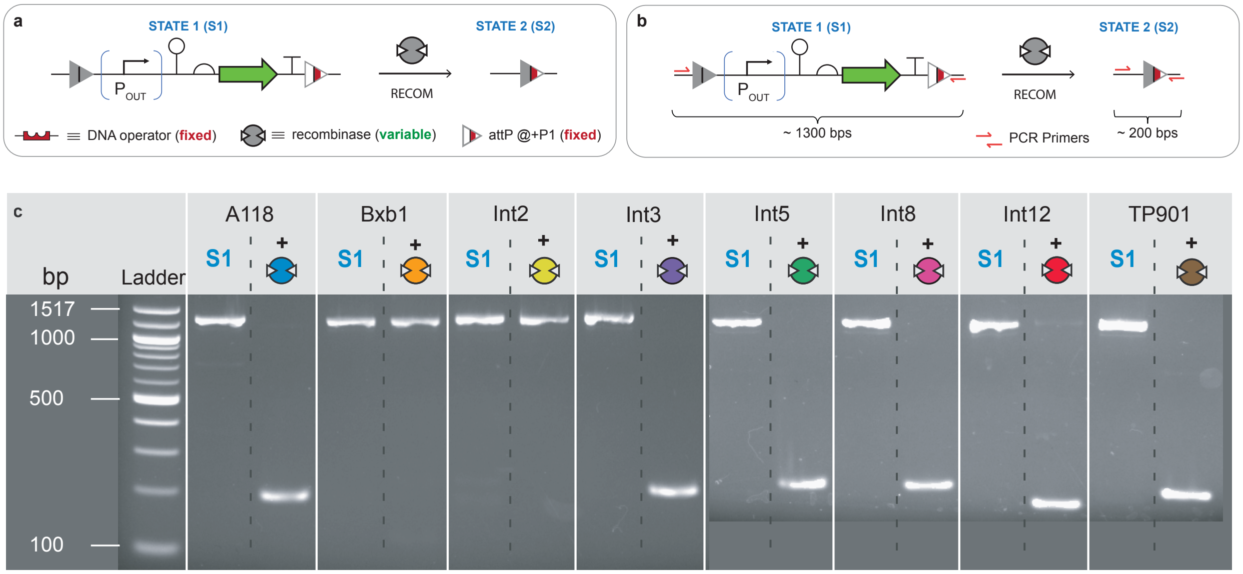

Supplementary Figure 4: Qualitative genotype of 8 recombinases paired with cognate interception deletion circuits. a Schematic of an interception deletion circuit, in which a constitutive promoter, ribozyme, RBS, and fluorescent protein GFP is nested between an aligned *attB* and *attP* pair (STATE 1). A recombinase matched to those att sites catalyzes recombination between *attB* and *attP*, resulting in deletion of GFP expression (STATE 2). An O^{ttg} operator is included at position P+1. **b** PCR primers that bind outside the reporter circuit can be used with gel electrophoresis to differentiate between an intact GFP circuit (state 1: 1,300 bp) and the recombined circuit (state 2: 200 bp). **c** Gel electrophoresis of colony PCR of the reporter circuit for cells transformed with only reporter and no recombinase (labeled "S1") versus cells transformed with reporter and recombinase (shown as "+ recombinase symbol"). This gel data is representative of three repeats of this experiment, each with similar results. The 100 bp DNA Ladder (NEB #N3271) is used as the reference. Source data are provided as a Source Data file.

Supplementary Fig. 5.

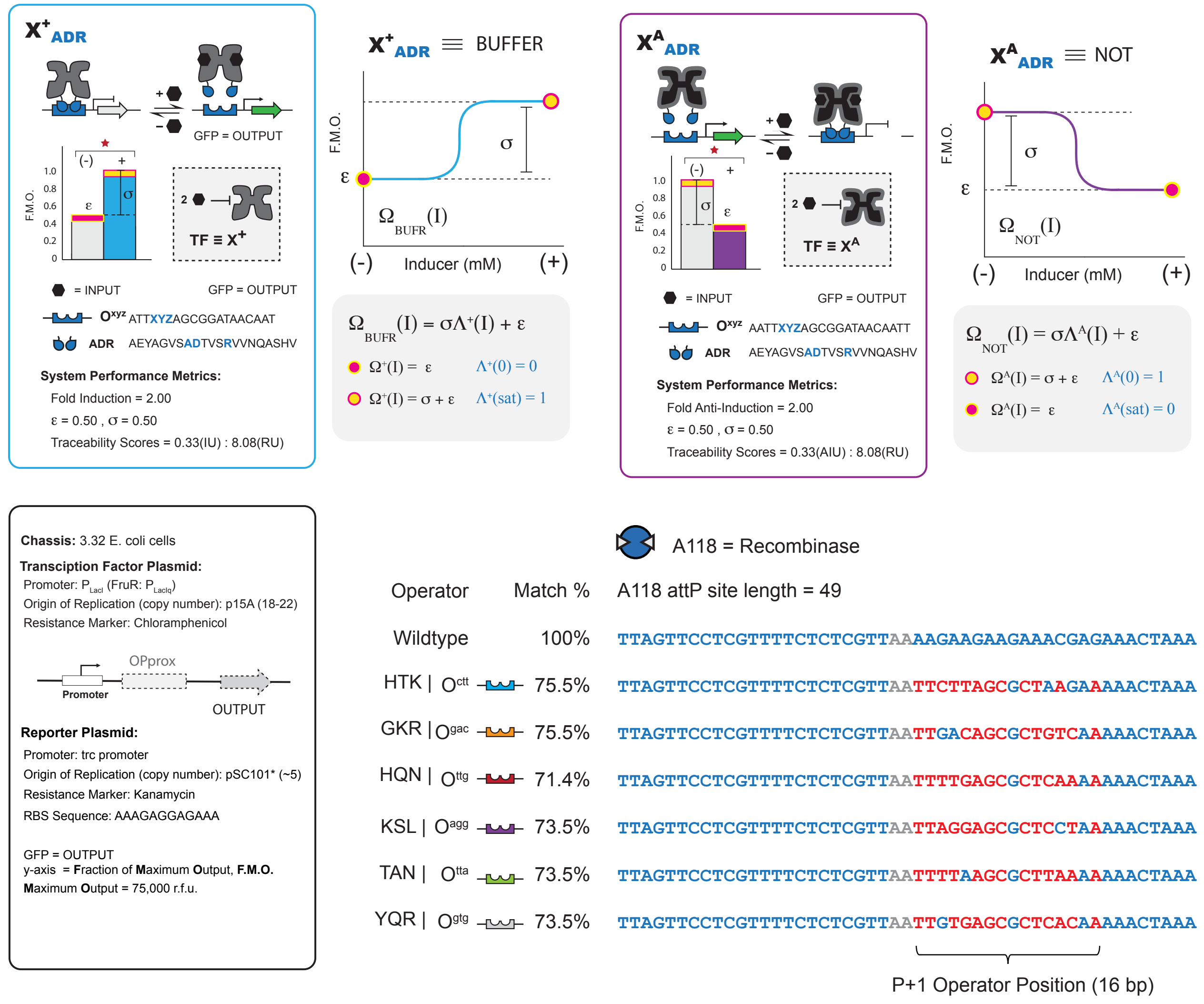

Supplementary Figure 5: Transcriptional repressor performance abstraction and sequence similarity between each operator substituted *attP* site relative to wildtype *attP*. At top left, the performance card for a general repressor (X^+) and an abstraction of its performance metrics to a logical BUFFER operation is shown. At top right, the performance card of a general anti-repressor (X^A) and an abstraction of its performance metrics to a logical NOT operation is shown. The metrology for a given single-INPUT single-OUTPUT (SISO) operation, we can model the induction profile for an experimentally verified SISO BUFFER operation *via* a coarse-grained binding function defined as

$$\Omega^+(I) = \sigma\Lambda^+(I) + \varepsilon \quad \textbf{Equation (1)}$$

where σ is a constant representing the maximum fluorescence – relative to basal expression of the OFF-state, $\Lambda^+(I)$ is a coarse-grained Hill function that can assume a value of 0 or 1, and ε represents fluorescence in the absence of inducer – *i.e.*, the OFF-state.

To model the performance of a given SISO NOT gate we used an analogous coarse-grained binding function – though for anti-repression – defined as

$$\Omega^A(I) = \sigma\Lambda^A(I) + \varepsilon \quad \textbf{Equation (2)}$$

where σ is a constant representing the maximum fluorescence, minus ligand – relative to basal expression of the OFF-state, $\Lambda^A(I)$ is a coarse-grained antithetical Hill-function for anti-repression where 0 INPUT corresponds to the ON-state, and 1 INPUT corresponds to the OFF-state, and ε represents fluorescence in the presents of inducer – *i.e.*, the OFF-state. This set of models was used to study performance prediction in Milner *et al.*[2]. At bottom left, the front of a general performance card is shown detailing the plasmids, PROXIMAL operator position, and chassis used to measure the TF performance data. At bottom right, the wildtype A118 *attP* site shown in blue (with central conserved region shown in grey) is compared to the *attP* sites used for interception with operators substituted at position P+1 (also see **Fig. 2j**). Nucleotides that are altered by the inclusion of the given operator are shown in red, highlighting the alteration to the *attP* site incurred by substituting each operator. The sequence similarity between each operator substituted *attP* site and wildtype is given as Match %, with lower scores indicating lower similarity.

Supplementary Fig. 6

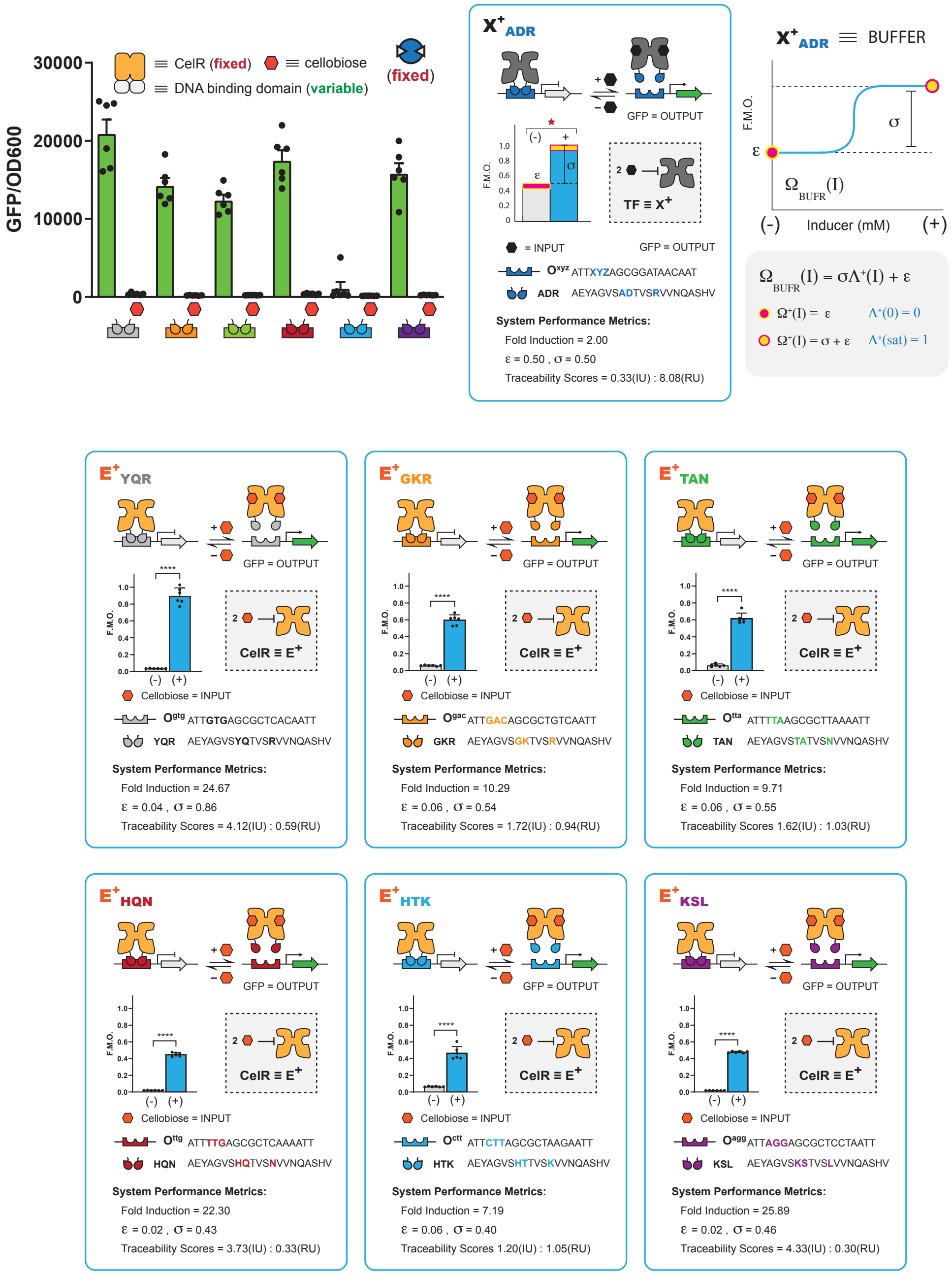

Supplementary Figure 6: Transcriptional repressor (E^+ variants) performance compared to repressor interception. Comparing interception performance of E^+ variants to transcriptional repression performance of those same E^+ variants. At top left, assay data is shown for intercepted (minus ligand) circuits versus deprotected (induced) circuits using the repressor E^+ across six different DNA-binding domain/operator pairs. At top right, the performance card for a general repressor (X^+) and an abstraction of its performance metrics to a logical BUFFER operation is shown – detailed description given in **Supplementary Figure 5**. Below, transcriptional repression performance cards for each of the E^+ variants tested for interception performance are given. Source data are provided as a Source Data file. Data represents the average of n = 6 biological replicates. Error bars correspond to the SEM of these measurements Source data are provided as a Source Data file. Error bars correspond to the SEM of these measurements. Welch's t-test between (-) inducer and (+) inducer groups was conducted were ****: p-value < 0.0001, *: p-value < 0.05, ns: p-value > 0.05.

Supplementary Fig. 7

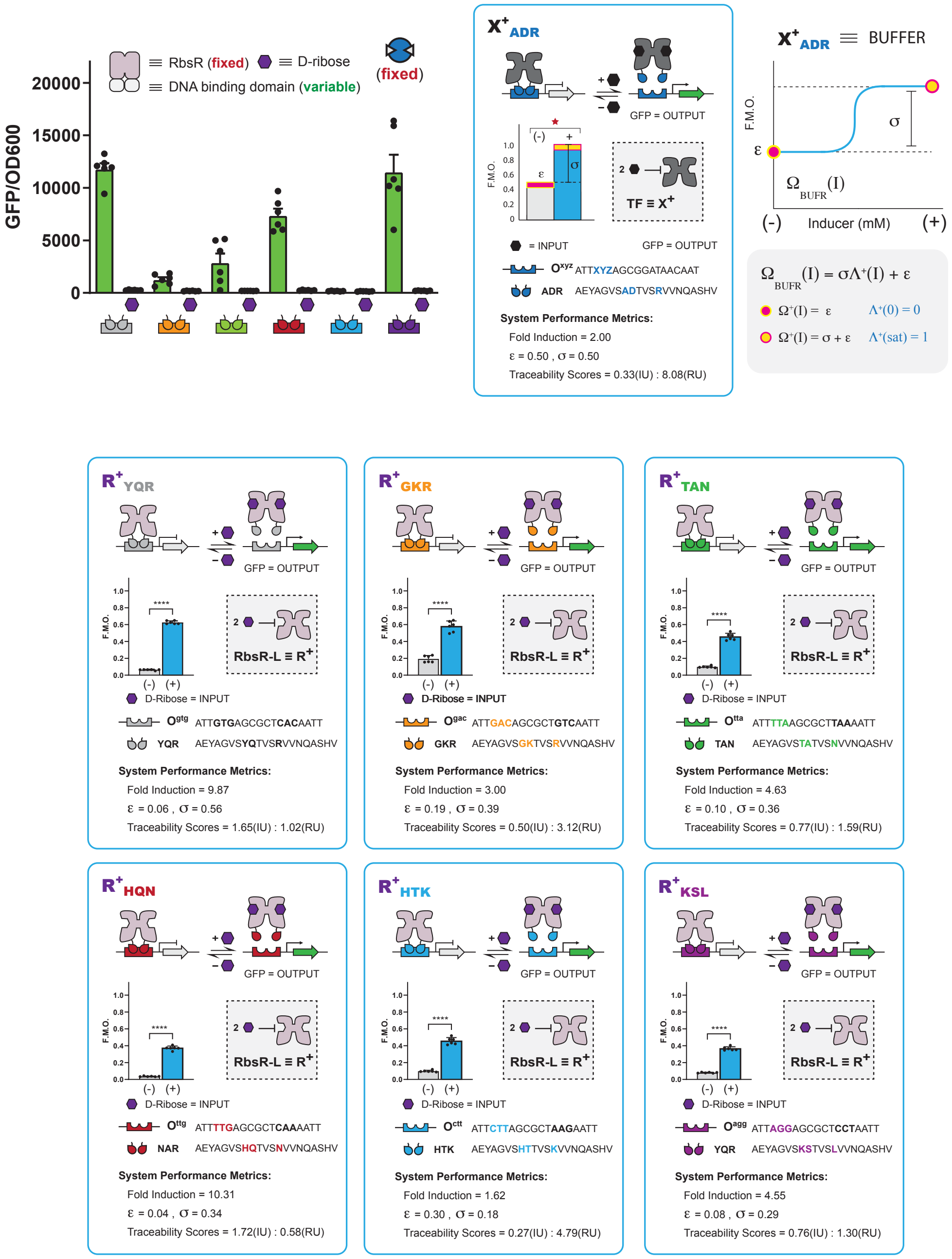

Supplementary Figure 7: Transcriptional repressor (R^+ variants) performance compared to repressor interception. Comparing interception performance of R^+ variants to transcriptional repression performance of those same R^+ variants. At top left, assay data is shown for intercepted (minus ligand) circuits versus deprotected (induced) circuits using the repressor R^+ across six different DNA-binding domain/operator pairs. At top right, the performance card for a general repressor (X^+) and an abstraction of its performance metrics to a logical BUFFER operation is shown – detailed description given in **Supplementary Figure 5**. Below, transcriptional repression performance cards for each of the R^+ variants tested for interception performance are given. Source data are provided as a Source Data file. Data represents the average of n = 6 biological replicates. Error bars correspond to the SEM of these measurements Source data are provided as a Source Data file. Welch's t-test between (-) inducer and (+) inducer groups was conducted were ****: p-value < 0.0001, *: p-value < 0.05, ns: p-value > 0.05.

Supplementary Fig. 8

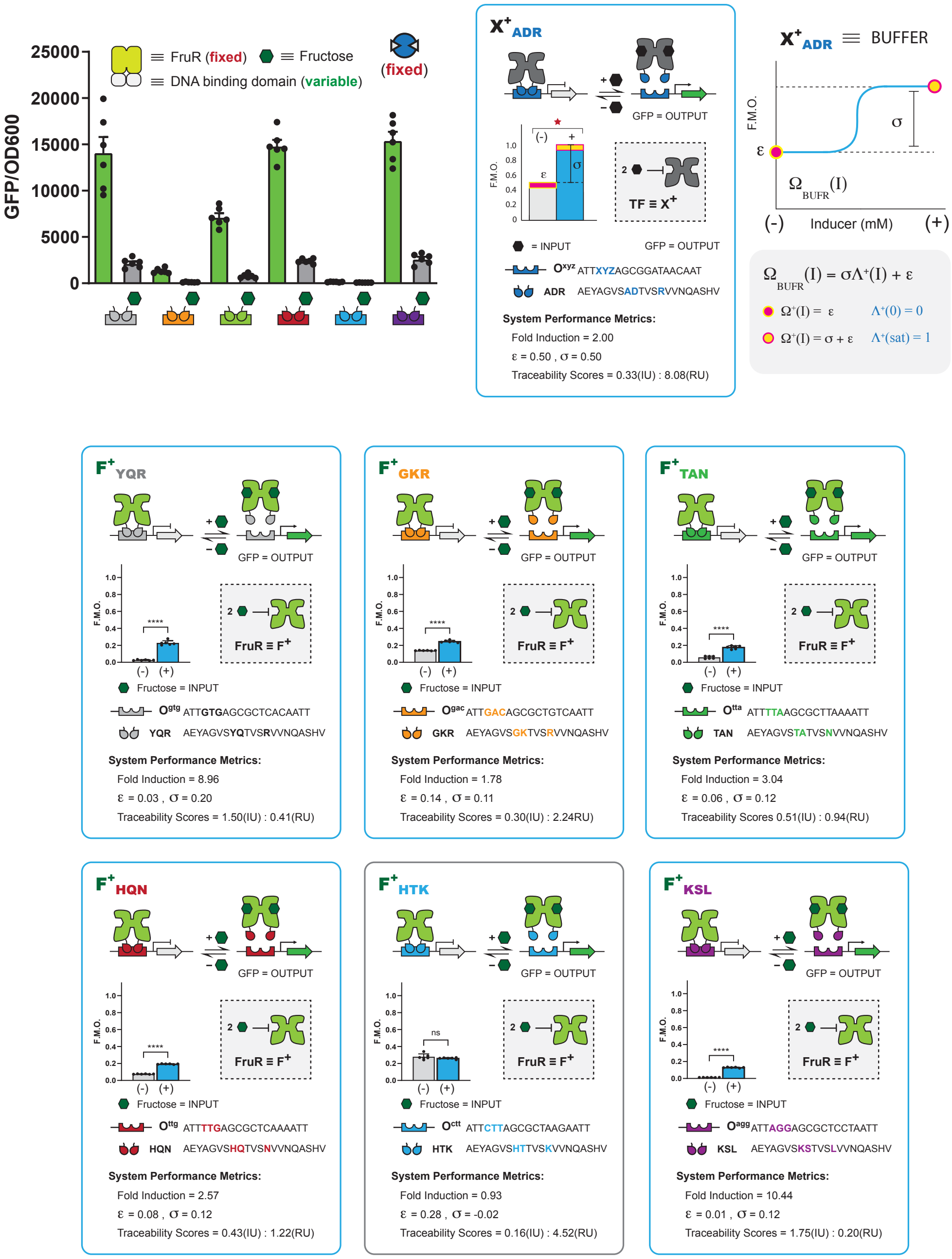

Supplementary Figure 8: Transcriptional repressor (F^+ variants) performance compared to repressor interception. Comparing interception performance of F^+ variants to transcriptional repression performance of those same F^+ variants. At top left, assay data is shown for intercepted (minus ligand) circuits versus deprotected (induced) circuits using the repressor F^+ across six different DNA-binding domain/operator pairs. At top right, the performance card for a general repressor (X^+) and an abstraction of its performance metrics to a logical BUFFER operation is shown – detailed description given in **Supplementary Figure 5**. Below, transcriptional repression performance cards for each of the F^+ variants tested for interception performance are given. Source data are provided as a Source Data file. Data represents the average of $n = 6$ biological replicates. Error bars correspond to the SEM of these measurements Source data are provided as a Source Data file. Welch's t-test between (-) inducer and (+) inducer groups was conducted were ****: p-value < 0.0001, *: p-value < 0.05, ns: p-value > 0.05.

Supplementary Fig. 9

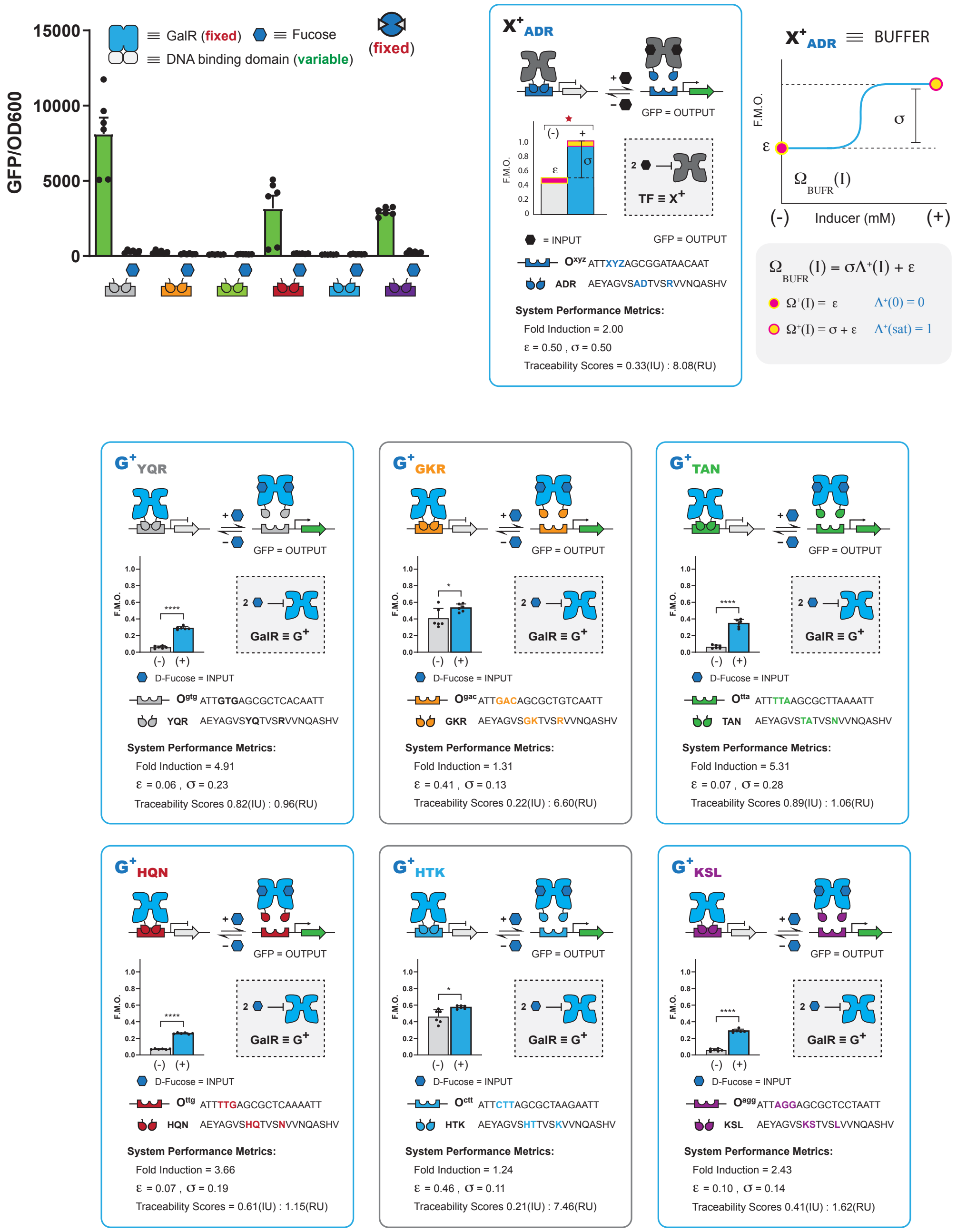

Supplementary Figure 9: Transcriptional repressor (G^+ variants) performance compared to repressor interception. Comparing interception performance of G^+ variants to transcriptional repression performance of those same G^+ variants. At top left, assay data is shown for intercepted (minus ligand) circuits versus deprotected (induced) circuits using the repressor G^+ across six different DNA-binding domain/operator pairs. At top right, the performance card for a general repressor (X^+) and an abstraction of its performance metrics to a logical BUFFER operation is shown – detailed description given in **Supplementary Figure 5**. Below, transcriptional repression performance cards for each of the G^+ variants tested for interception performance are given. Source data are provided as a Source Data file. Data represents the average of n = 6 biological replicates. Error bars correspond to the SEM of these measurements Source data are provided as a Source Data file. Welch's t-test between (-) inducer and (+) inducer groups was conducted were ****: p-value < 0.0001, *: p-value < 0.05, ns: p-value > 0.05.

Supplementary Fig. 10

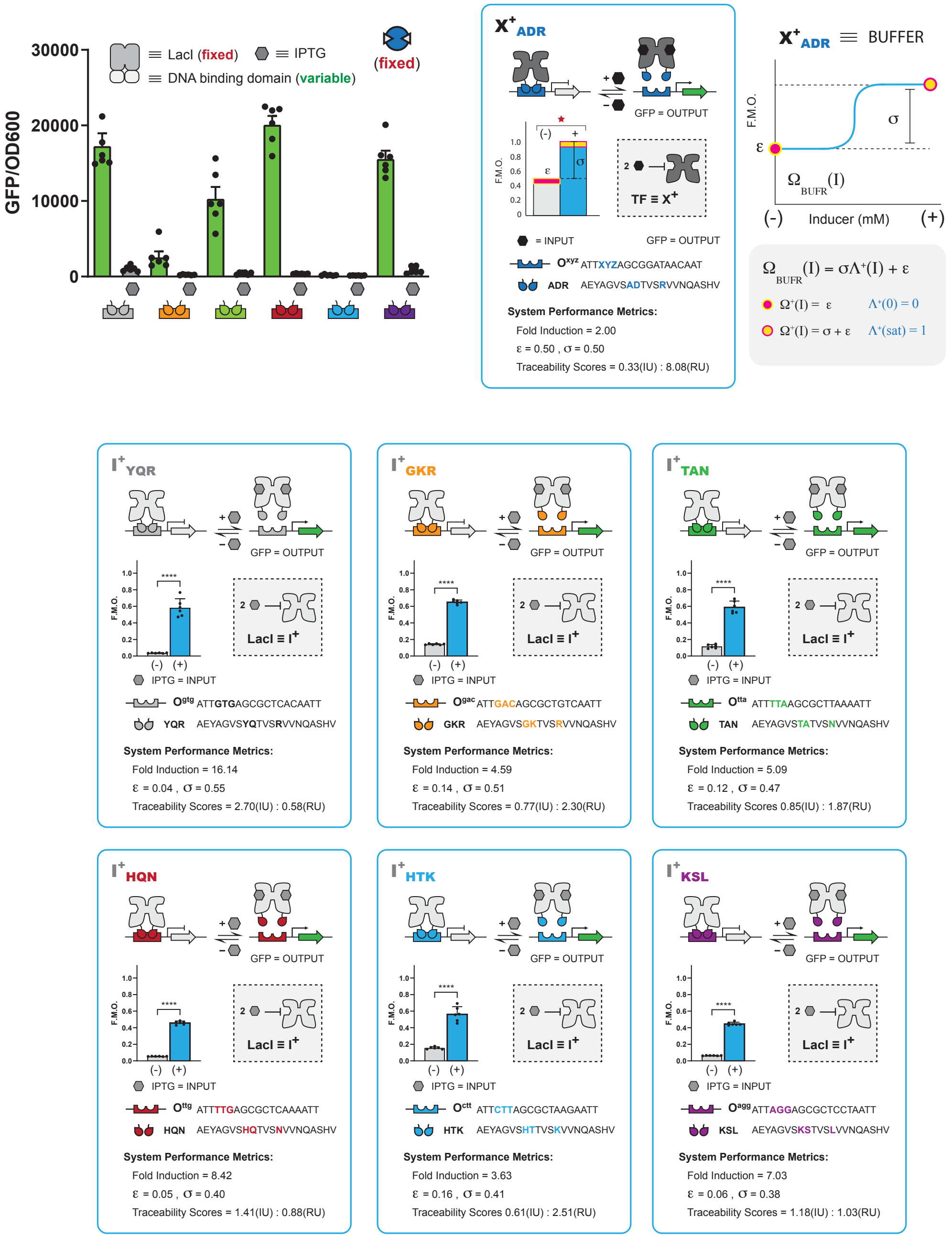

Supplementary Figure 10: Transcriptional repressor (I^+ variants) performance compared to repressor interception. Comparing interception performance of I^+ variants to transcriptional repression performance of those same I^+ variants. At top left, assay data is shown for intercepted (minus ligand) circuits versus deprotected (induced) circuits using the repressor I^+ across six different DNA-binding domain/operator pairs. At top right, the performance card for a general repressor (X^+) and an abstraction of its performance metrics to a logical BUFFER operation is shown – detailed description given in **Supplementary Figure 5**. Below, transcriptional repression performance cards for each of the I^+ variants tested for interception performance are given. Source data are provided as a Source Data file. Data represents the average of n = 6 biological replicates. Error bars correspond to the SEM of these measurements Source data are provided as a Source Data file. Welch's t-test between (-) inducer and (+) inducer groups was conducted were ****: p-value < 0.0001, *: p-value < 0.05, ns: p-value > 0.05.

Supplementary Fig. 11

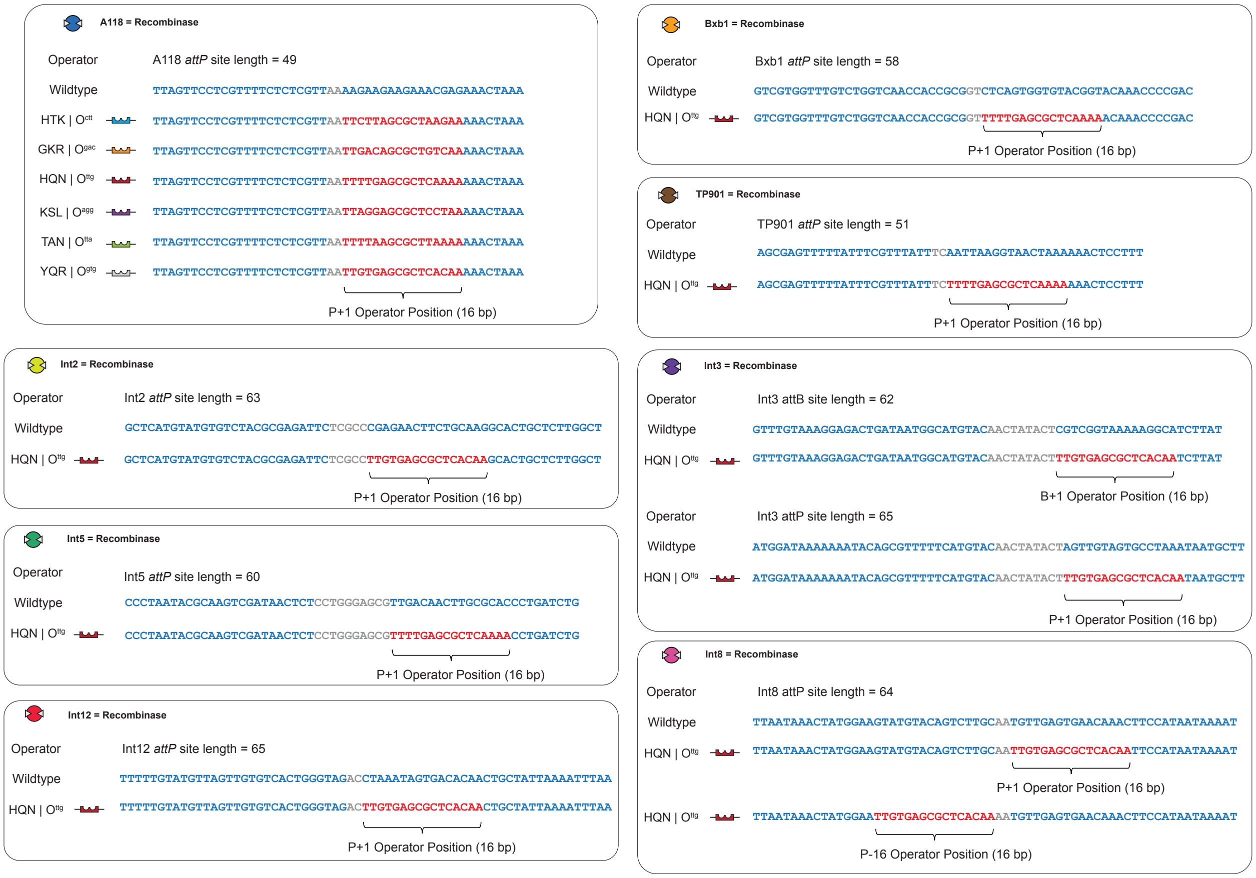

Supplementary Figure 11: Detailed sequences for substituted *attP* sites. Nucleotides that are altered by the substitution with operator DNA are shown in red, highlighting the alteration to the *attP* site incurred by substituting with a given operator.

Supplementary Fig. 12

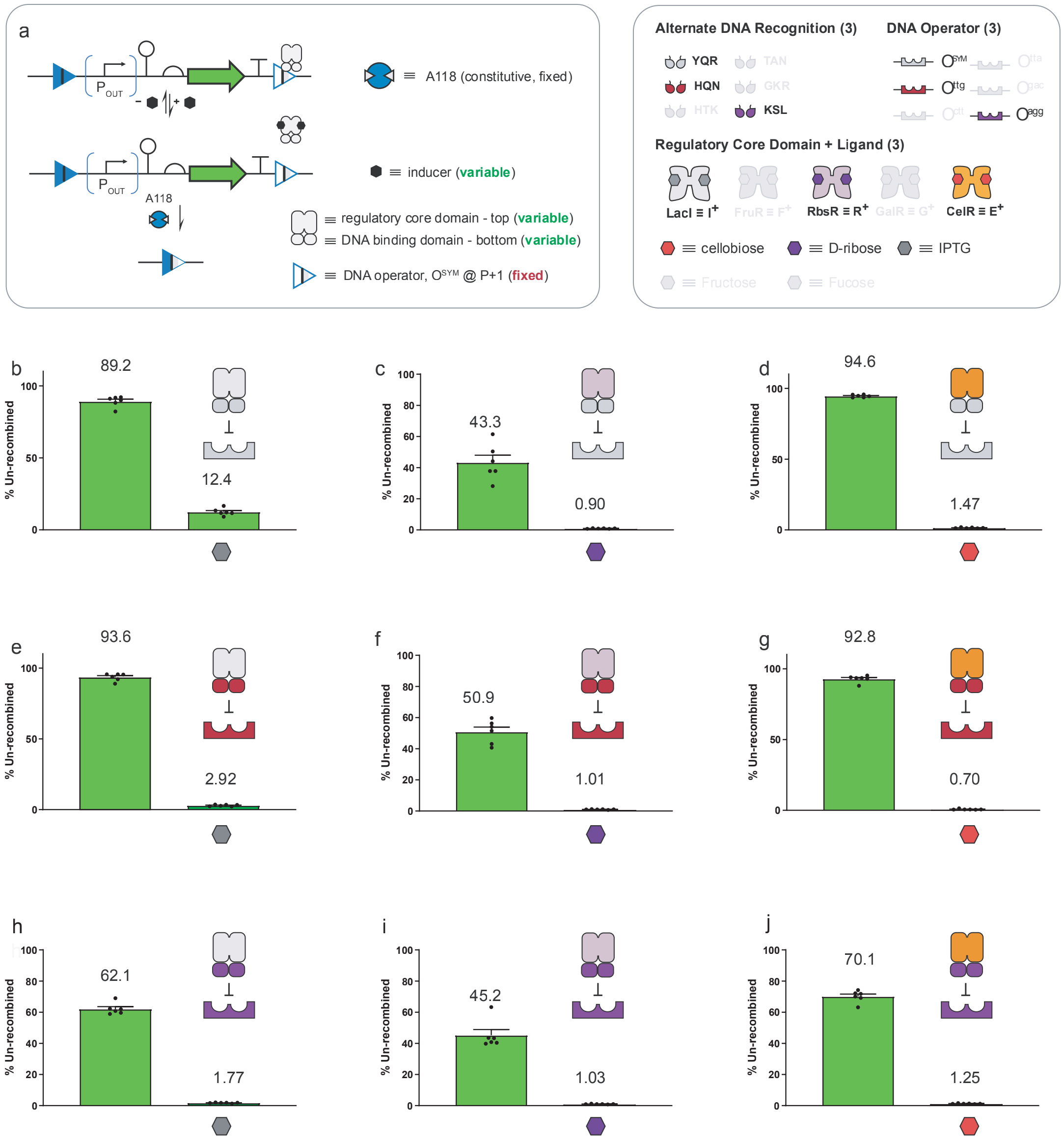

Supplementary Figure 12: Flow cytometry of select interception circuits. a Inset at left is a schematic summarizing the genetic construct used to assess A118 recombinase interception with variable repressors directed at different operators placed in the P+1 position. Inset at right is the subset of modular TF components used for flow cytometry analysis of interception performance. **b-j** Flow cytometry analysis of different TFs intercepting A118 *via* different operators in the P+1 position. The distribution of cells in the two possible states of fluorescent protein expression after growth in medium without inducers is shown. Source data are provided as a Source Data file. Data in **b-j** represent the average of n = 6 biological replicates. Error bars correspond to the SEM of these measurements.

Supplementary Fig. 13

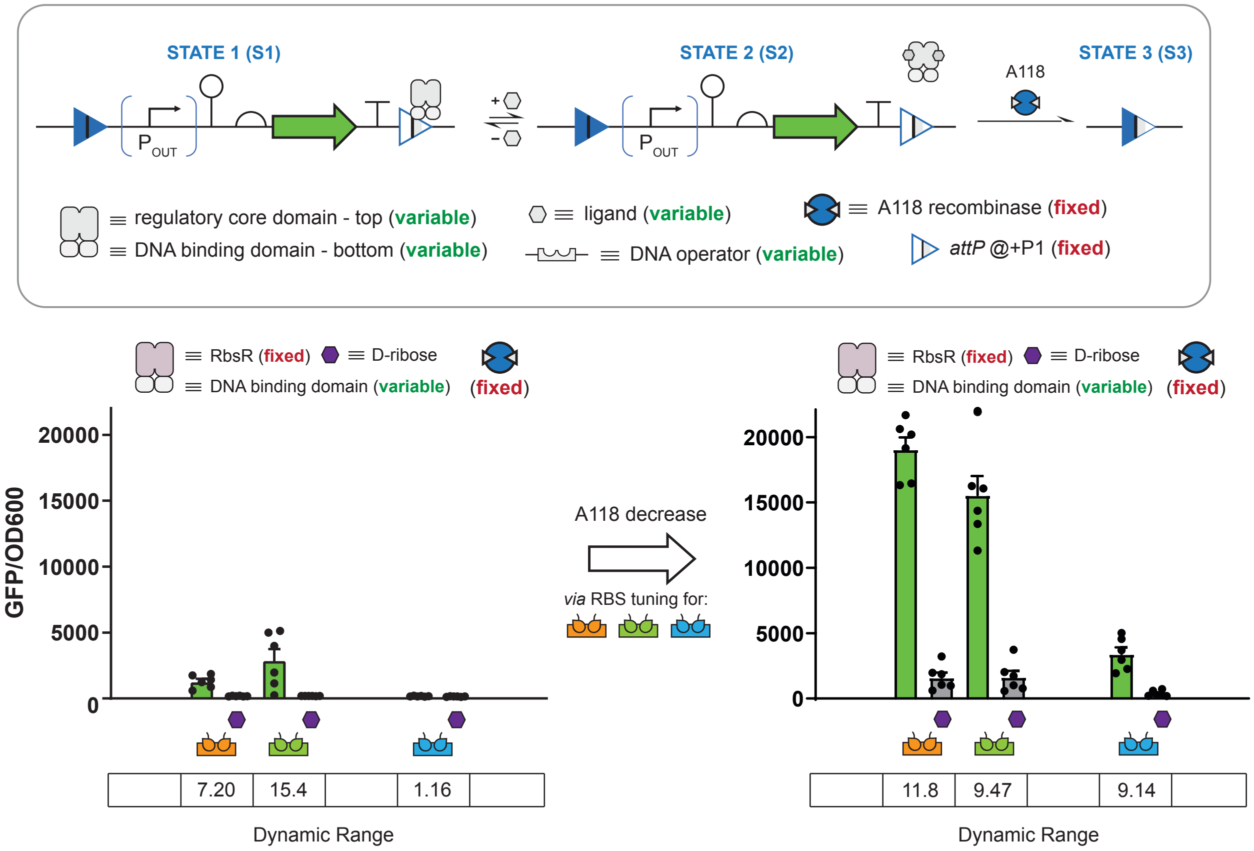

Supplementary Figure 13: Ribosome binding site (RBS) tuning of A118 for three R$^+$ ADR. (top) A schematic summarizing the mechanism and genetic construct (deletion circuit) used to assess A118 recombinase interception with variable repressors directed at different operators placed in the P+1 position. (bottom left) Assay data collected under moderate conditions (see **Supplementary Note 2**) for R$^+$, also given in **Fig. 3**. (bottom right) Assay data for three ADR/operator pairs, GKR/O^{gac}, TAN/O^{tta}, and HTK/O^{ctt} following A118 expression modification *via* a prescribed RBS library. Source data are provided as a Source Data file. Data represents the average of $n = 6$ biological replicates. Error bars correspond to the SEM of these measurements.

Supplementary Fig. 14

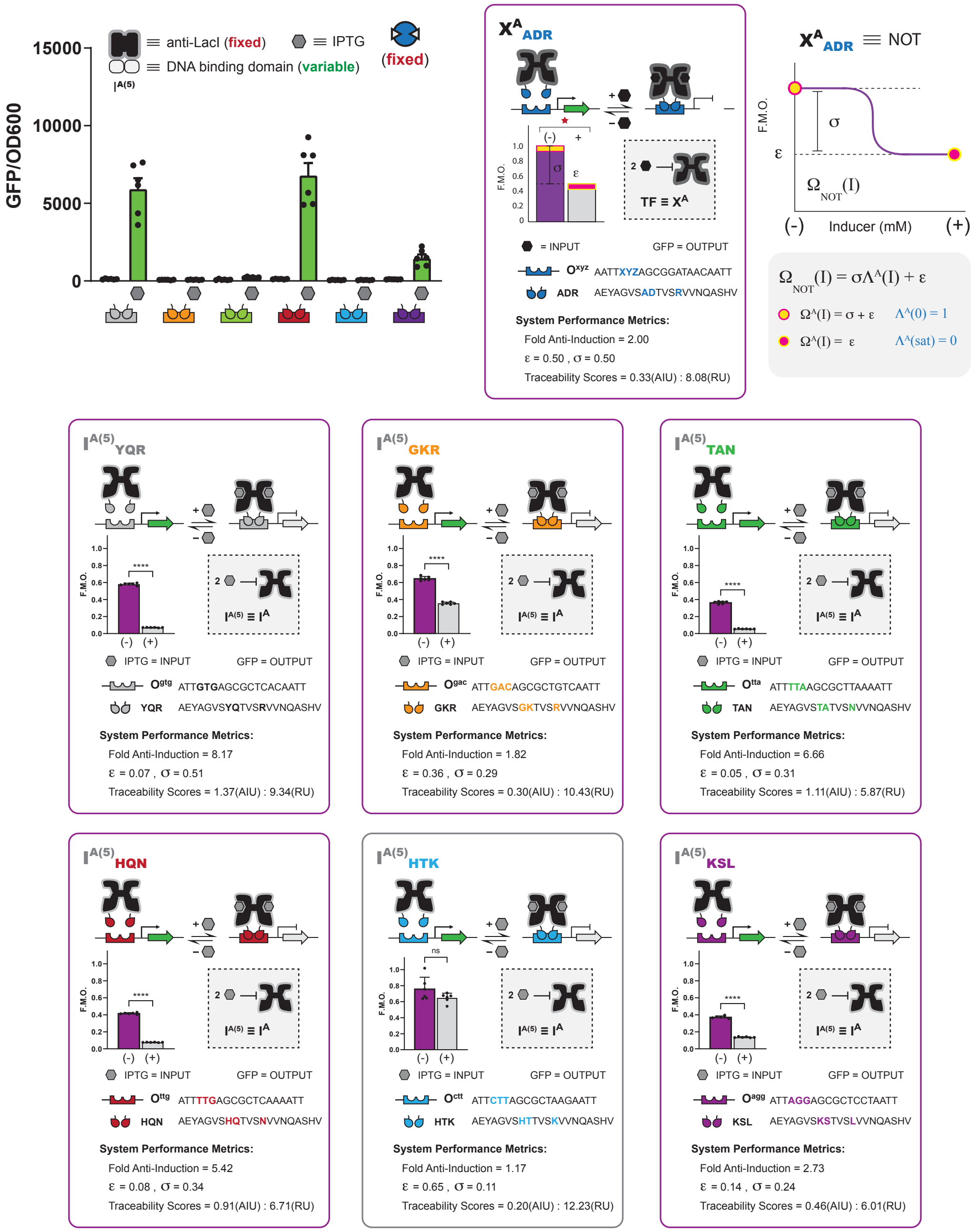

Supplementary Figure 14: Transcriptional anti-repressor (I^A variants) performance compared to anti-repressor interception. Comparing interception performance of $I^{A(5)}$ variants to transcriptional anti-repression performance of those same $I^{A(5)}$ variants. At top left, assay data is shown for intercepted (induced) circuits versus deprotected (minus ligand) circuits using the anti-repressor $I^{A(5)}$ across six different DNA-binding domain/operator pairs. At top right, the performance card for a general anti-repressor (X^A) and an abstraction of its performance metrics to a logical NOT operation is shown – detailed description given in **Supplementary Figure 5**. Below, transcriptional anti-repression performance cards for each of the $I^{A(5)}$ variants tested for interception performance are given. Source data are provided as a Source Data file. Data represents the average of $n = 6$ biological replicates. Error bars correspond to the SEM of these measurements Source data are provided as a Source Data file. Welch's t-test between (-) inducer and (+) inducer groups was conducted were ****: p-value < 0.0001, *: p-value < 0.05, ns: p-value > 0.05.

Supplementary Fig. 15

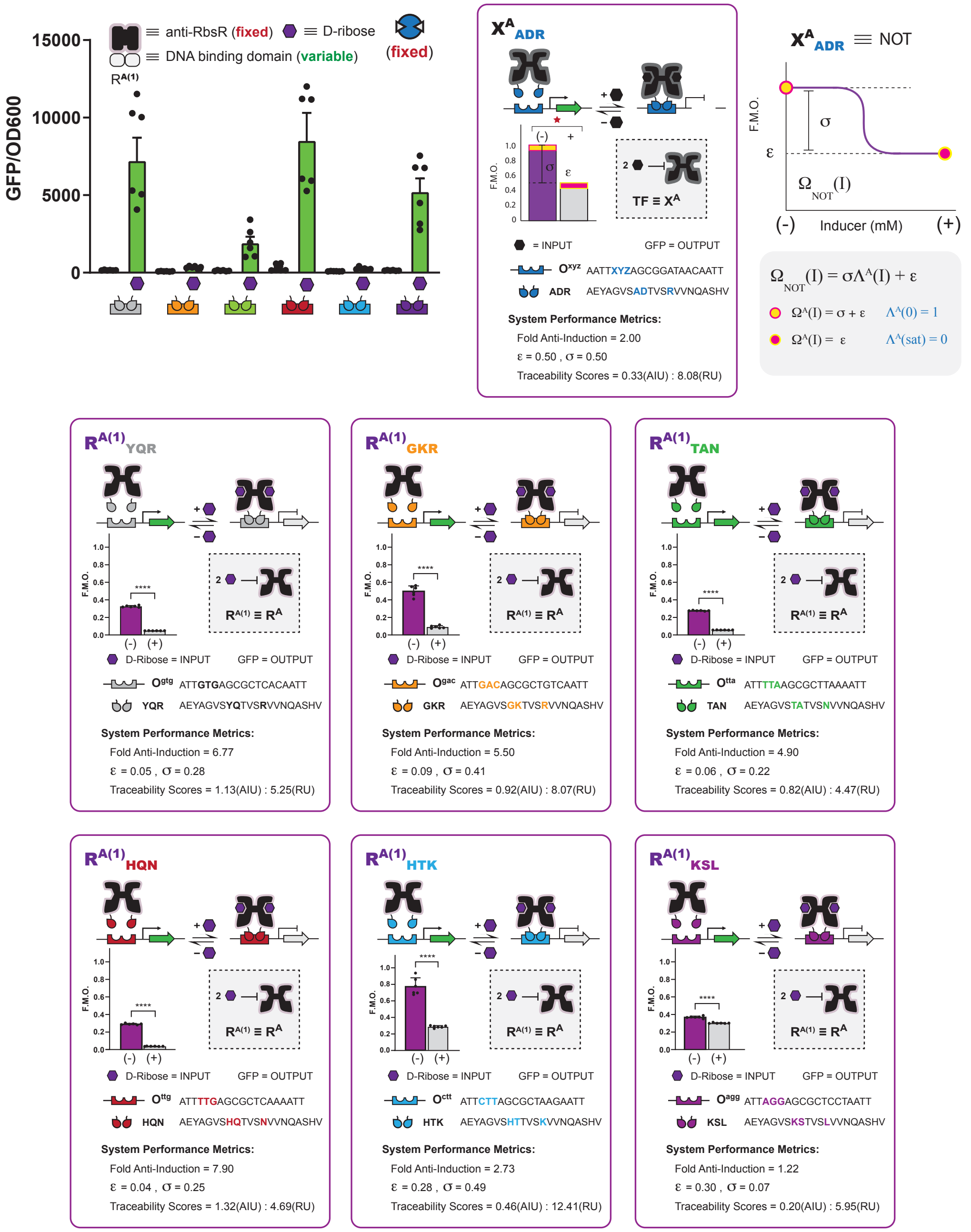

Supplementary Figure 15: Transcriptional anti-repressor (R^A variants) performance compared to anti-repressor interception. Comparing interception performance of $R^{A(1)}$ variants to transcriptional anti-repression performance of those same $R^{A(1)}$ variants. At top left, assay data is shown for intercepted (induced) circuits versus deprotected (minus ligand) circuits using the anti-repressor $R^{A(1)}$ across six different DNA-binding domain/operator pairs. At top right, the performance card for a general anti-repressor (X^A) and an abstraction of its performance metrics to a logical NOT operation is shown – detailed description given in **Supplementary Figure 5**. Below, transcriptional anti-repression performance cards for each of the $R^{A(1)}$ variants tested for interception performance are given. Source data are provided as a Source Data file. Data represents the average of $n = 6$ biological replicates. Error bars correspond to the SEM of these measurements Source data are provided as a Source Data file. Welch's t-test between (-) inducer and (+) inducer groups was conducted were ****: p-value < 0.0001, *: p-value < 0.05, ns: p-value > 0.05.

Supplementary Fig. 16

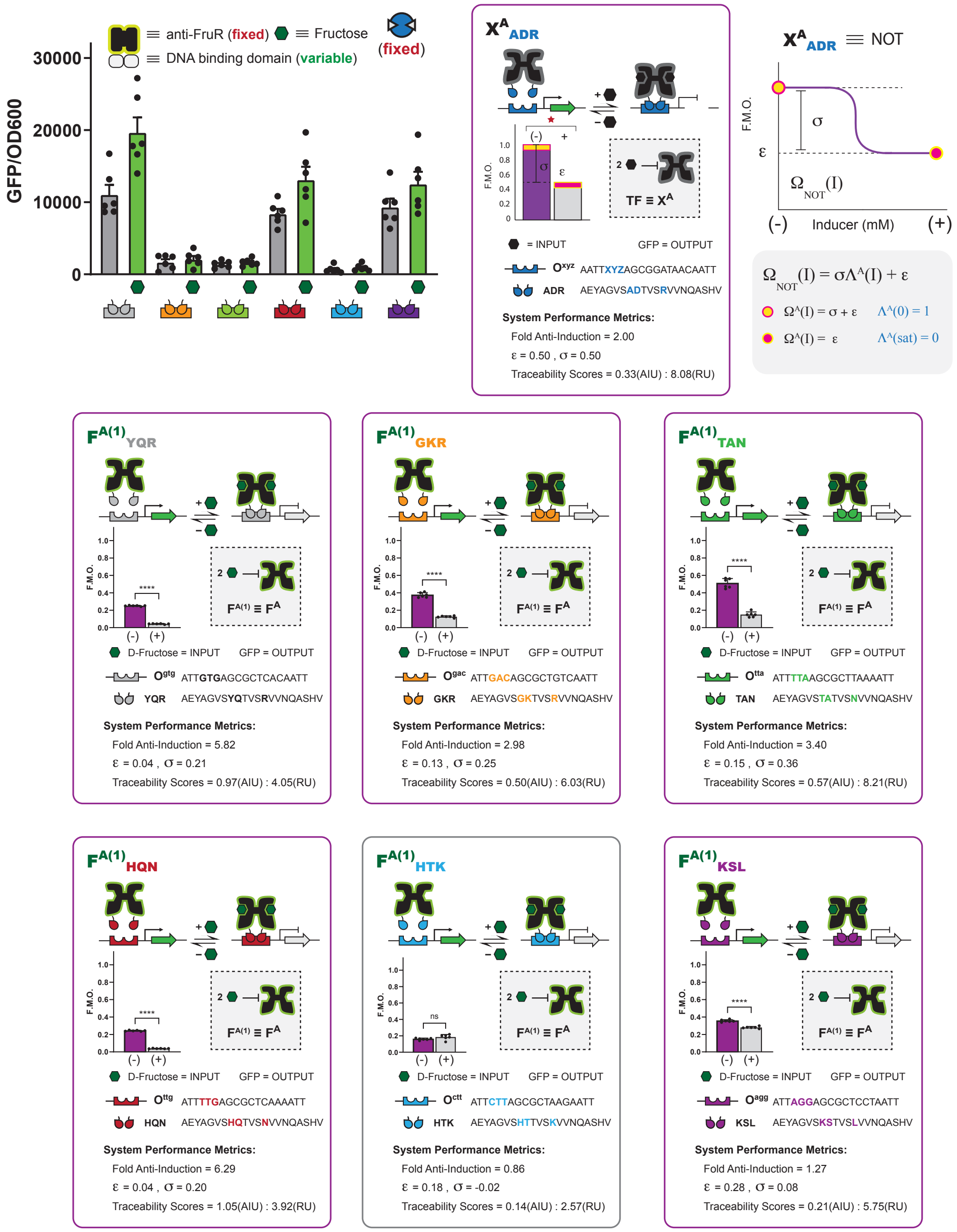

Supplementary Figure 16: Transcriptional anti-repressor (F^A variants) performance compared to anti-repressor interception. Comparing interception performance of $F^{A(1)}$ variants to transcriptional anti-repression performance of those same $F^{A(1)}$ variants. At top left, assay data is shown for intercepted (induced) circuits versus deprotected (minus ligand) circuits using the anti-repressor $F^{A(1)}$ across six different DNA-binding domain/operator pairs. At top right, the performance card for a general anti-repressor (X^A) and an abstraction of its performance metrics to a logical NOT operation is shown – detailed description given in **Supplementary Figure 5**. Below, transcriptional anti-repression performance cards for each of the $F^{A(1)}$ variants tested for interception performance are given. Source data are provided as a Source Data file. Data represents the average of *n* = 6 biological replicates. Error bars correspond to the SEM of these measurements Source data are provided as a Source Data file. Welch's t-test between (-) inducer and (+) inducer groups was conducted were ****: p-value < 0.0001, *: p-value < 0.05, ns: p-value > 0.05.

Supplementary Fig. 17

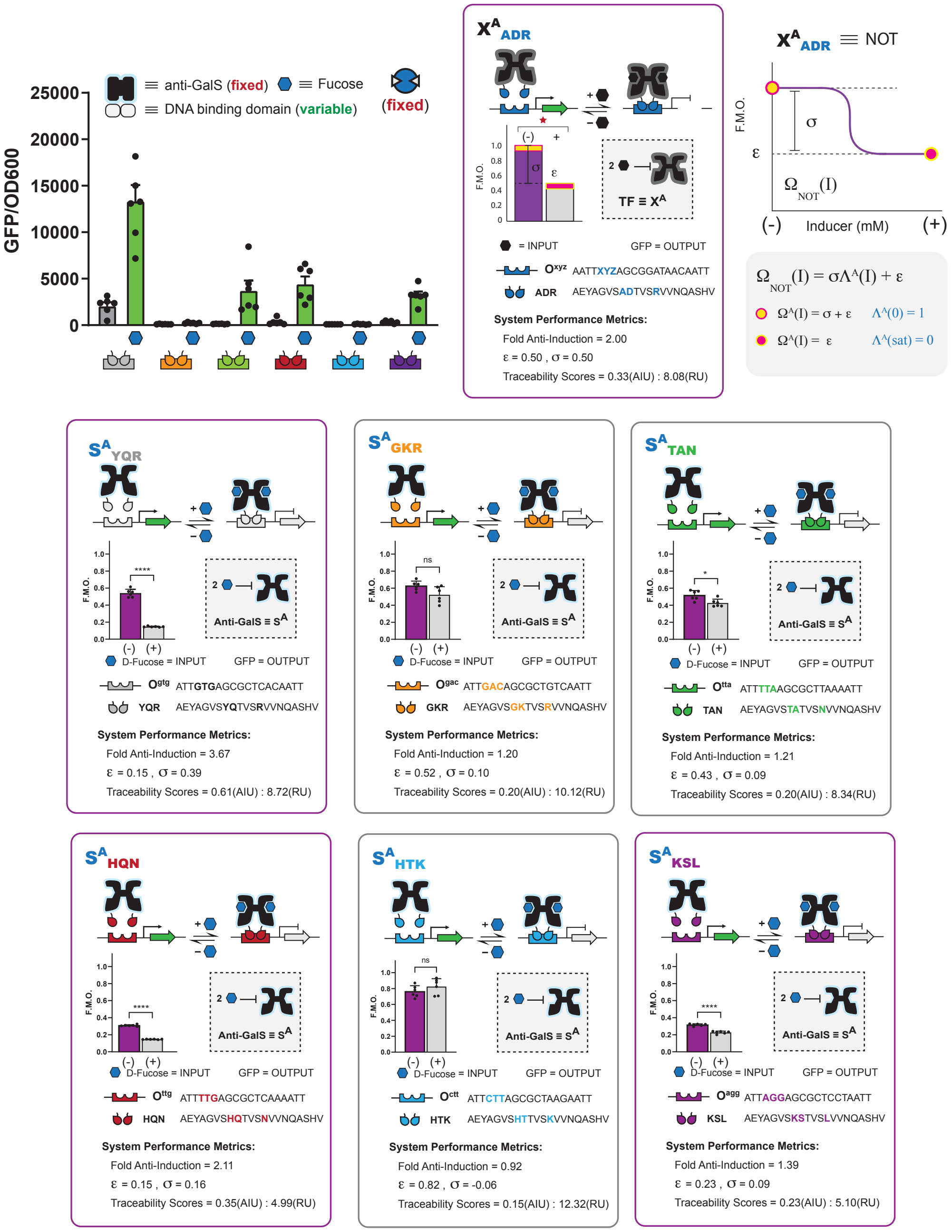

Supplementary Figure 17: Transcriptional anti-repressor (S^A variants) performance compared to anti-repressor interception. Comparing interception performance of $S^{A(1)}$ variants to transcriptional anti-repression performance of those same $S^{A(1)}$ variants. At top left, assay data is shown for intercepted (induced) circuits versus deprotected (minus ligand) circuits using the anti-repressor $S^{A(1)}$ across six different DNA-binding domain/operator pairs. At top right, the performance card for a general anti-repressor (X^A) and an abstraction of its performance metrics to a logical NOT operation is shown – detailed description given in **Supplementary Figure 5**. Below, transcriptional anti-repression performance cards for each of the $S^{A(1)}$ variants tested for interception performance are given. Source data are provided as a Source Data file. Data represents the average of $n = 6$ biological replicates. Error bars correspond to the SEM of these measurements Source data are provided as a Source Data file. Welch's t-test between (-) inducer and (+) inducer groups was conducted were ****: p-value < 0.0001, *: p-value < 0.05, ns: p-value > 0.05.

Supplementary Fig. 18

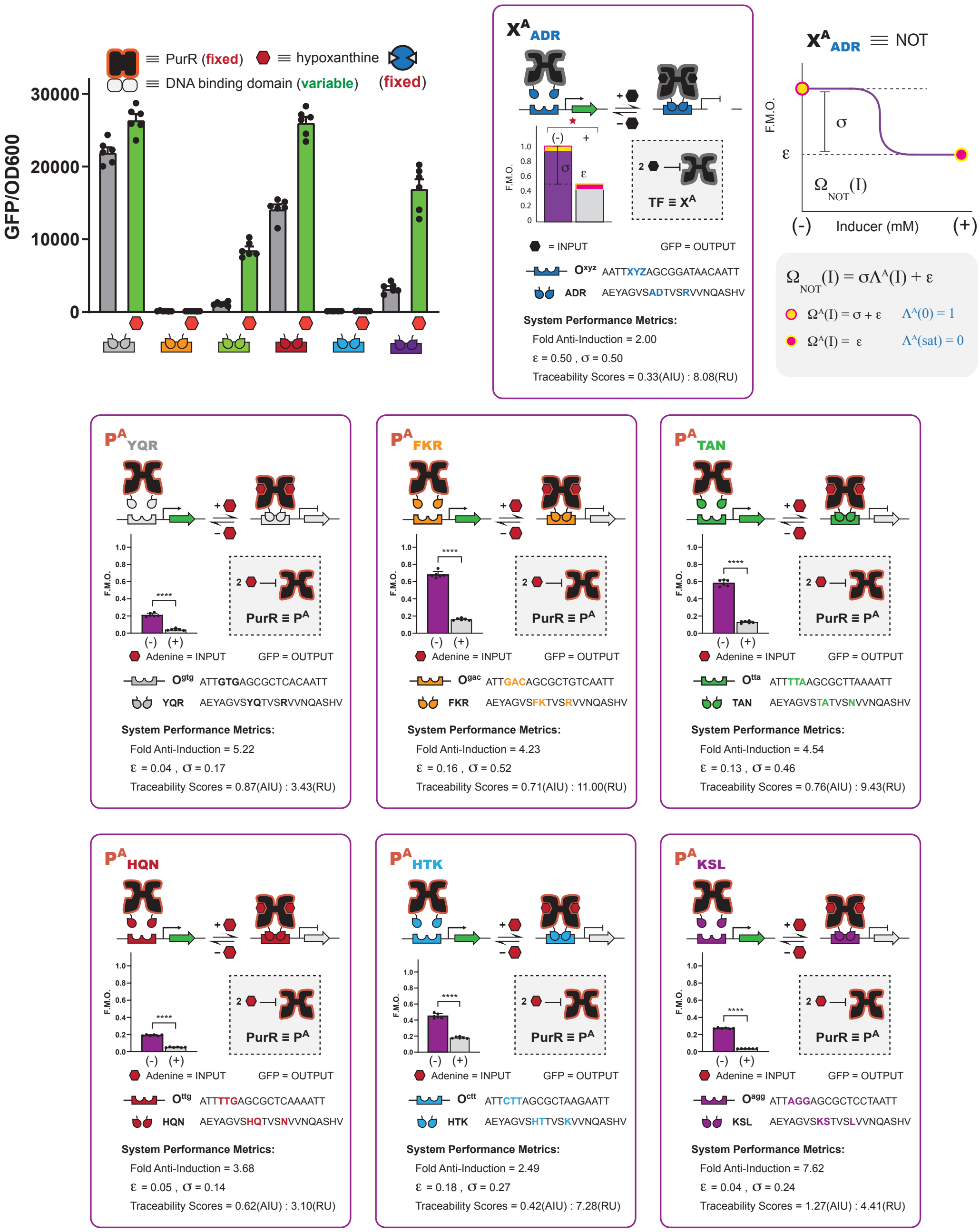

Supplementary Figure 18: Transcriptional anti-repressor (P^A variants) performance compared to anti-repressor interception. Comparing interception performance of P^A variants to transcriptional anti-repression performance of those same P^A variants. At top left, assay data is shown for intercepted (induced) circuits versus deprotected (minus ligand) circuits using the anti-repressor P^A across six different DNA-binding domain/operator pairs. At top right, the performance card for a general anti-repressor (X^A) and an abstraction of its performance metrics to a logical NOT operation is shown – detailed description given in **Supplementary Figure 5**. Below, transcriptional anti-repression performance cards for each of the P^A variants tested for interception performance are given. Source data are provided as a Source Data file. Data represents the average of $n = 6$ biological replicates. Error bars correspond to the SEM of these measurements Source data are provided as a Source Data file. Welch's t-test between (-) inducer and (+) inducer groups was conducted were ****: p-value < 0.0001, *: p-value < 0.05, ns: p-value > 0.05.

Supplementary Fig. 19

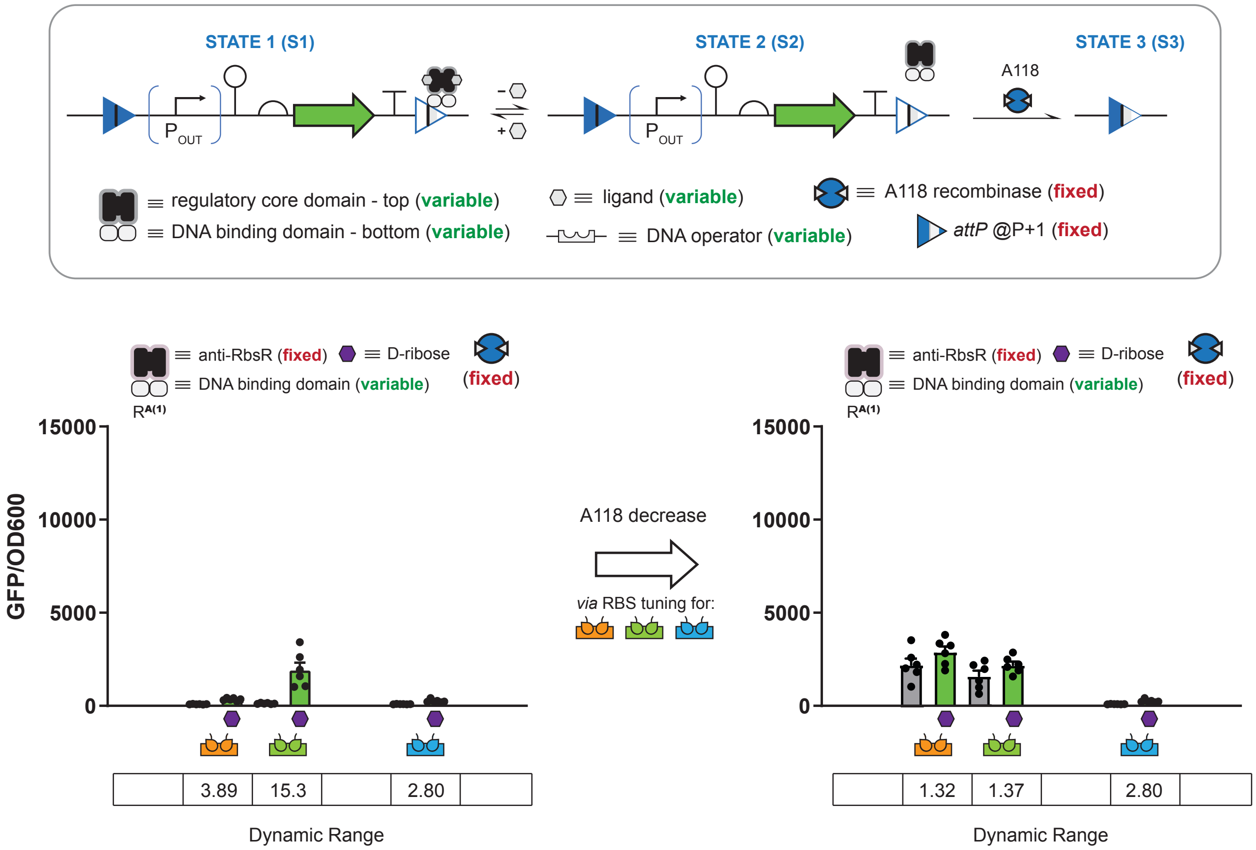

Supplementary Figure 19: Ribosome binding site (RBS) tuning of A118 for three $R^{A(1)}$ ADR. (top) Schematic summarizing the mechanism and genetic construct (deletion circuit) used to assess A118 recombinase interception for different anti-repressors directed at different operators placed in the P+1 position. (bottom left) Assay data collected under moderate conditions (see **Supplementary Note 2**) for $R^{A(1)}$, also given in **Fig. 4**. (bottom right) Assay data for three ADR/operator pairs, GKR/O^{gac}, TAN/O^{tta}, and HTK/O^{ctt} following A118 expression modification *via* RBS library. Source data are provided as a Source Data file. Data represents the average of $n = 6$ biological replicates. Error bars correspond to the SEM of these measurements.

Supplementary Fig. 20

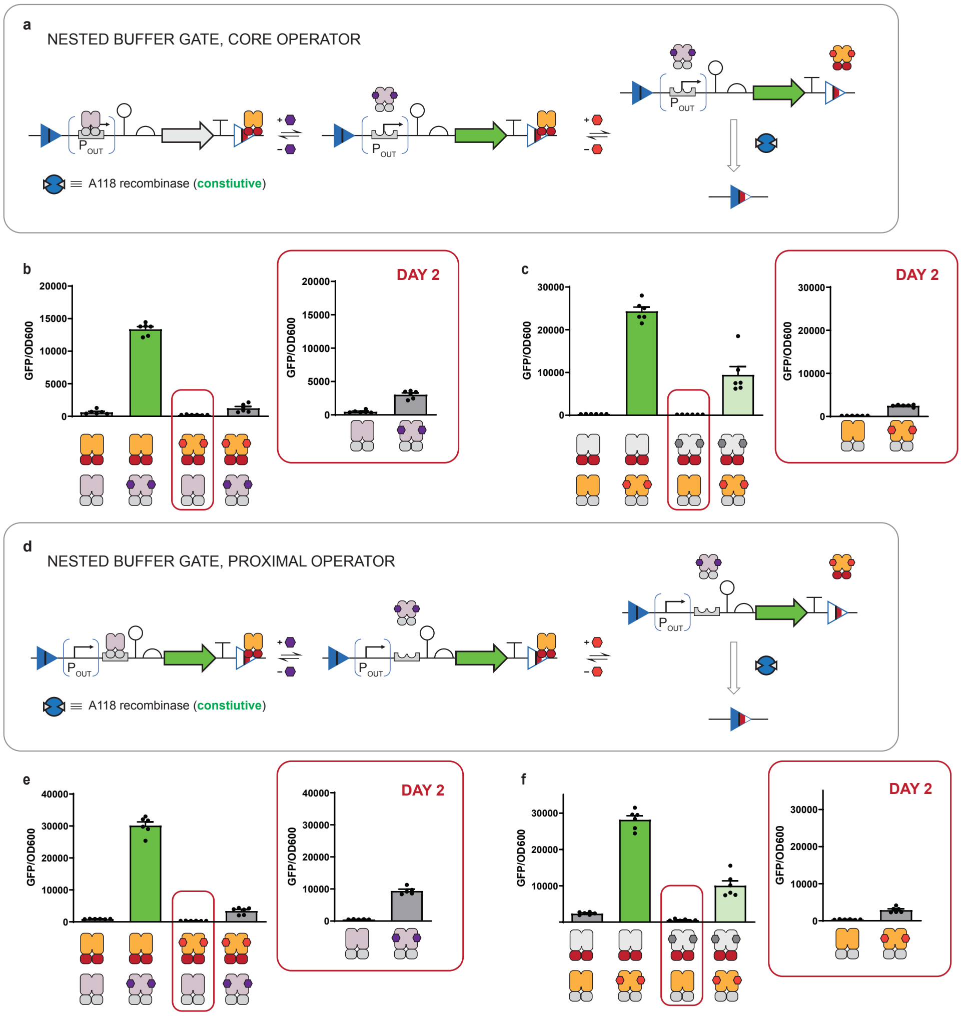

Supplementary Figure 20: Nested BUFFER logic paired with interception memory. a Schematic of a BUFFER logic gate consisting of GFP regulated by R^{+}_{YQR} directed to the O^{gtg} operator in the CORE position nested within an A118-mediated deletion circuit, intercepted *via* E^{+}_{HQN} and cognate operator O^{ttg} at position P+1. A118 is constitutively expressed in all assays. When R^{+}_{YQR} is induced, GFP OUTPUT is expressed – *i.e.*, the circuit operates as a simple BUFFER gate. When E^{+}_{HQN} is induced, the circuit is deprotected and A118 recombines the att sites, deleting the nested BUFFER gate. **b** Assay data for the given circuit controlled by R^{+}_{YQR} and intercepted by E^{+}_{HQN} under different inducer conditions. Inducing only R^{+}_{YQR} results in the production of the GFP OUTPUT – *i.e.*, the circuit functions as a BUFFER logic operation. Inducing E^{+}_{HQN} enables A118-mediated deletion of the nested logic circuit, observed as mitigated GFP fluorescence. The assay condition with only cellobiose added, boxed in red, was diluted and grown in fresh minimal media for 20 additional hours with and without ribose. The data in the red box shows the resulting phenotypes, confirming deletion. **c** Assay data for this circuit controlled by an alternate TF set – *i.e.*, BUFFER logic controlled by E^{+}_{YQR} and interception by I^{+}_{HQN}. Inducing only E^{+}_{YQR} results in GFP OUTPUT – *i.e.*, functioning as a simple BUFFER logical operation. Inducing I^{+}_{HQN} enables A118-mediated deletion of the nested logic circuit, resulting in mitigated GFP fluorescence. The assay condition with only IPTG added, boxed in red, was diluted and grown in fresh minimal media for 20 additional hours with and without cellobiose. The data in the red box shows the resulting phenotypes. **d-f** are additional nested logical operations *via* alternate transcription factors, *i.e.,* with the BUFFER operator O^{gtg} in the PROXIMAL position. Source data are provided as a Source Data file. Data represents the average of n = 6 biological replicates. Error bars correspond to the SEM of these measurements.

Supplementary Fig. 21

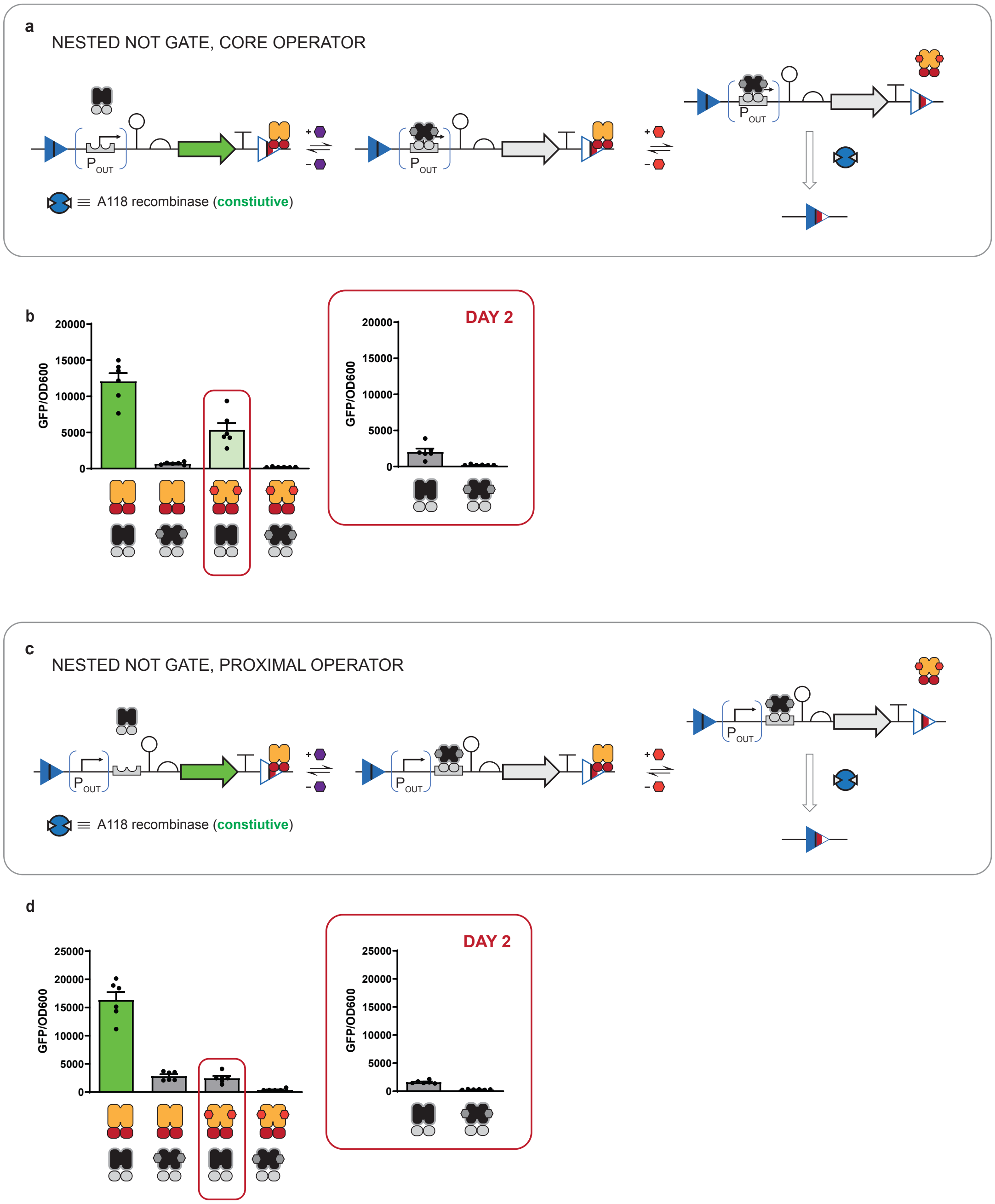

Supplementary Figure 21: Nested NOT logic paired with interception memory. a Schematic of a NOT logic gate consisting of GFP regulated by $I^{A(9)}{}_{YQR}$ directed to the O^{gtg} operator in the CORE position nested within an A118 deletion circuit that is intercepted by $E^{+}{}_{HQN}$ at the O^{ttg} operator placed at the P+1 position. **b** Assay data for the given circuit controlled by $I^{A(9)}{}_{YQR}$ and intercepted by $E^{+}{}_{HQN}$. Inducing only $I^{A(9)}{}_{YQR}$ resulted in mitigated GFP OUTPUT – *i.e.*, the circuit objectively functions as a NOT logical operation. Inducing $E^{+}{}_{HQN}$ enables A118-mediated deletion of the nested circuit, resulting in abrogated NOT logic. The assay condition with only cellobiose added, boxed in red, was diluted and grown in fresh minimal media for 20 additional hours with and without IPTG. The data in the red box shows the resulting phenotypes, confirming circuit deletion. **c-d** similar to **a-b**, however with the NOT operator O^{gtg} in the PROXIMAL position. Source data are provided as a Source Data file. Data represents the average of n = 6 biological replicates. Error bars correspond to the SEM of these measurements.

Supplementary Fig. 22

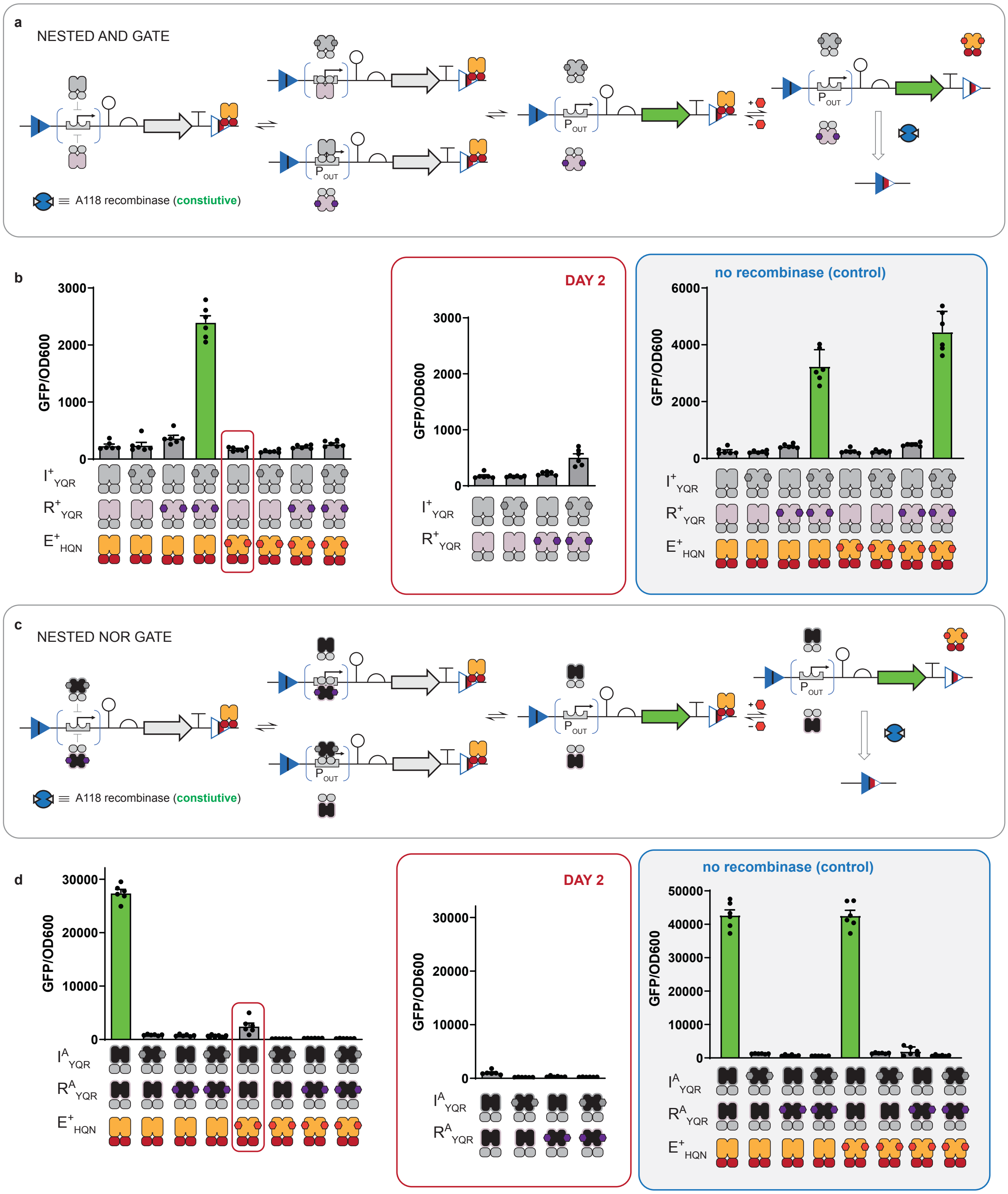

Supplementary Figure 22: Interception synthetic memory with nested AND / NOR Boolean Logic. We constructed interception memory circuits with nested 2-INPUT logic. Namely, we constructed a circuit with a nested AND gate (see **a-b**) and a separate circuit with a nested NOR gate (see **c-d**). In the first iteration of the 2-INPUT circuit, the nested AND logic with interception was functional, requiring 2-INPUTs to produce GFP – unless the attachment site was deprotected upon the addition of cellobiose see **a-b**. Moreover, in the absence of recombinase the circuit performed as a simple AND gate – independent of the presence of cellobiose (see **b** inset blue box). Likewise, the nested NOR iteration of the interception circuit was also functional – with a synonymous control feature regulating the deletion memory operation (see **c-d**). Namely, the protected circuit only produced GFP in the absence of both IPTG and ribose. However, upon the addition of cellobiose the circuit was deleted (see **c-d**). **a** Genetic schematic and mechanism of a transcriptional AND gate nested within a memory interception circuit (coded for deletion), cognate to the A118 recombinase. A118 and the regulating TFs (I^{+}_{YQR}, R^{+}_{YQR}, and E^{+}_{HQN}) are expressed constitutively. I^{+}_{YQR} and R^{+}_{YQR} regulate GFP expression by binding to an O^{gtg} operator in the promoter's core position, and E^{+}_{HQN} intercepts A118 function at an O^{ttg} promoter placed at the P+1 position. From left to right, the response to different inducers (INPUTs) is shown. **b** Assay data for the circuit shown in **a**. Cells exposed to cellobiose (*i.e.*, post deletion) are boxed in red. Post deletion, cells were diluted 1:200 and grown in fresh minimal media for 20 additional hours with and without the transcriptional logic INPUTS IPTG and ribose to demonstrate deletion memory. Data in the red box labeled DAY 2 shows the resulting phenotypes. To the right (of the red box) boxed in blue is the control data for this circuit transformed with the TF-expression plasmid and without the recombinase expression plasmid. **c** Genetic schematic of a transcriptional NOR gate nested within a memory interception circuit (coded for deletion), cognate to the A118 recombinase. A118 and the regulating TFs ($I^{A(9)}_{YQR}$, $R^{A(2)}_{YQR}$, and E^{+}_{HQN}) are expressed constitutively. $I^{A(9)}_{YQR}$ and $R^{A(2)}_{YQR}$ regulate GFP expression by binding to an O^{gtg} operator in the promoter's core position, and E^{+}_{HQN} intercepts A118 function at an O^{ttg} promoter placed at the P+1 position. **d** Assay data for the circuit shown in **c**. Cells exposed to cellobiose (*i.e.*, post deletion) are boxed in red. Post deletion, cells were diluted 1:200 and grown in fresh minimal media for 20 additional hours with and without the transcriptional logic INPUTS IPTG and ribose to demonstrate deletion memory. Data in the red box shows the resulting phenotypes. To the right (of the red box) boxed in blue is the control data for this circuit transformed with the TF-expression plasmid and without the recombinase expression plasmid. Source data are provided as a Source Data file. Data in **b** and **d** represent the average of n = 6 biological replicates. Error bars correspond to the SEM of these measurements.

Supplementary Fig. 23

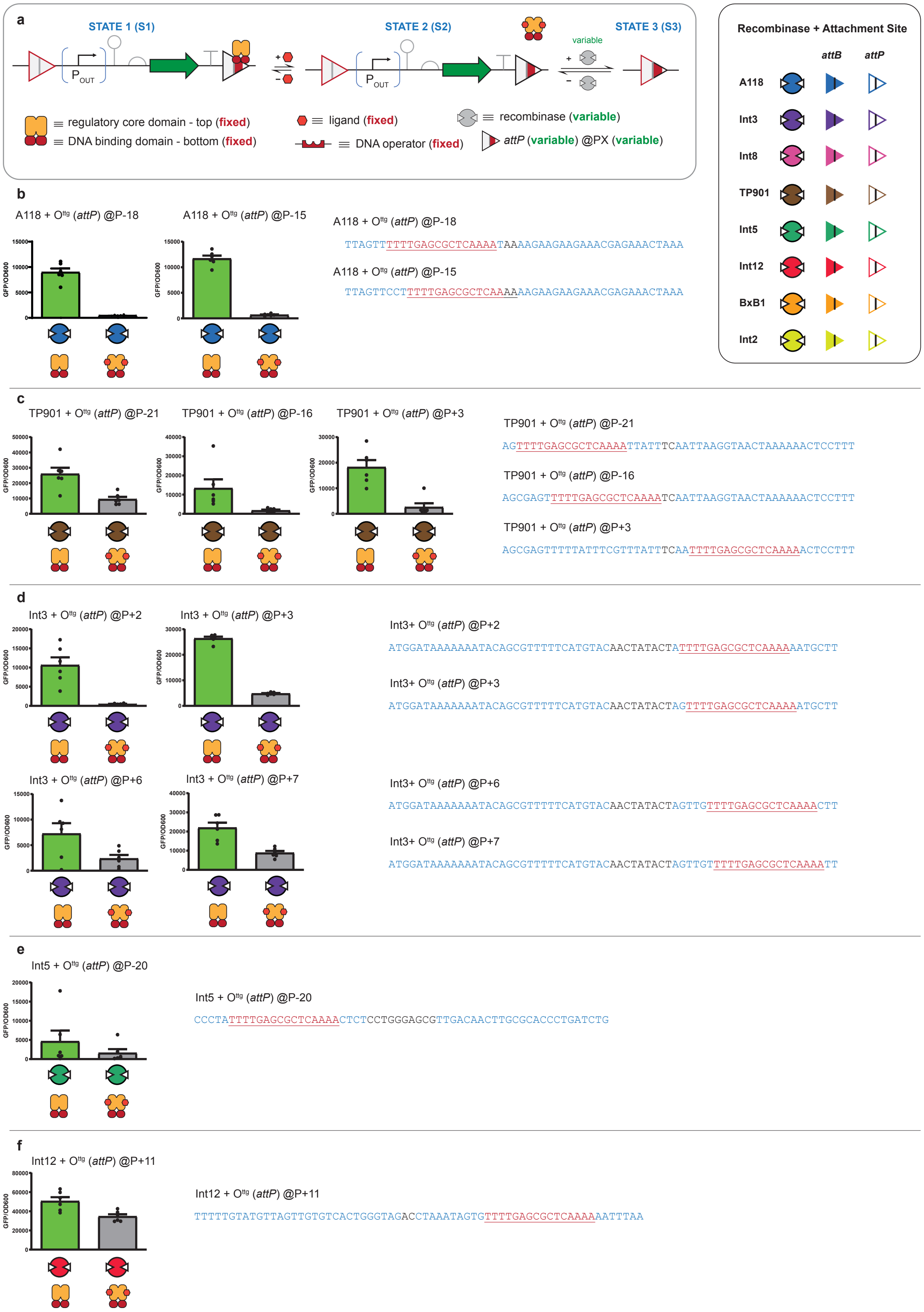

Supplementary Figure 23: Additional operator positions, interception *via* A118, TP901, Int3, Int5, and Int12. a A schematic summarizing the genetic construct used to assess interception of five different recombinases by E^{+}_{HQN} directed at O^{ttg} *via* variable operator positions. The relevant recombinase and E^{+} HQN are constitutively expressed in all cases. In STATE 1, E^{+}_{HQN} binding at the operator blocks recombinase function, protecting the circuit from deletion. Inducing the repressor brings the circuit to STATE 2, where the recombinase can access the *attP* site to recombine the circuit, bringing the circuit to STATE 3. **b** Alternate operator positions that facilitate interception *via* the A118 recombinase. Inset text displays the genetic edits made to include O^{ttg} operators (shown in red and underlined) at those *attP* positions (shown in blue, with central conserved region shown in black). **c** Alternate interception operator positions for the recombinase TP901. **d** Alternate operator positions for the recombinase Int3. **e** Alternate operator position for the recombinase Int5. **f** Alternate operator position for the recombinase Int12. Source data are provided as a Source Data file. Data in **b-f** represent the average of n = 6 biological replicates. Error bars correspond to the SEM of these measurements.

Supplementary Fig. 24

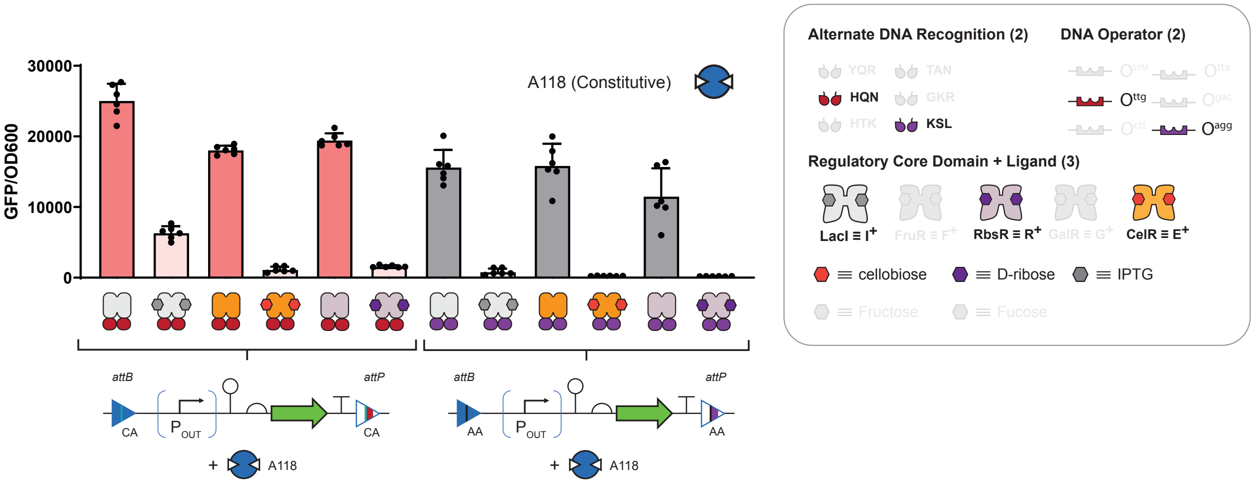

Supplementary Figure 24: Interception performance with different central dinucleotides. At left, assay data is shown for different TFs intercepting two A118 deletion circuits, one with CA central dinucleotides and an O^{ttg} operator at the P+1 position (data shown in pink) and one with AA central dinucleotides and an O^{agg} operator at the P+1 position (data shown in gray). I^{+}_{HQN}, E^{+}_{HQN}, and R^{+}_{HQN} were tested for interception of the CA circuit at O^{ttg} both with and without inducer in the presence of constitutive A118 expression. I^{+}_{KSL}, E^{+}_{KSL}, and R^{+}_{KSL} were tested for interception of the AA circuit at O^{agg} both with and without inducer in the presence of constitutive A118 expression. (inset) Modular TF components used in this study. Source data are provided as a Source Data file. Data represents the average of n = 6 biological replicates. Error bars correspond to the SEM of these measurements.

Supplementary Fig. 25

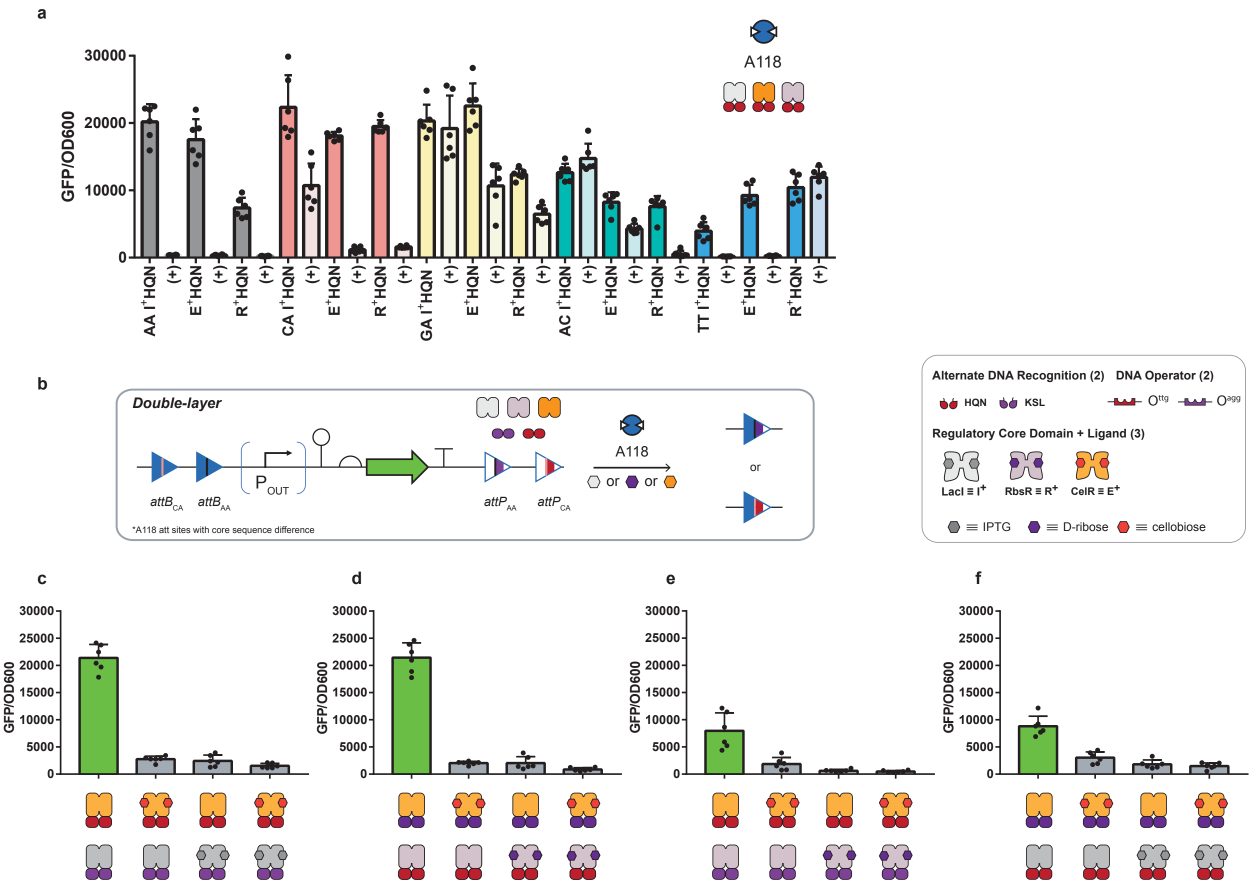

Supplementary Figure 25: Double-layer deletion circuit with two orthogonal attachment sites. Recombinase activities are measured with varying the central conserved region of the attachment sites. **a** Interception was tested on reporter circuits shown in **Fig. 8a** for central conserved regions AA, CA, GA, AC, and TT with O^{ttg} operator inserted at the P+1 position. Each pair of bars on the plot show results for a different repressor (I^{+}_{HQN}, E^{+}_{HQN}, and R^{+}_{HQN}) intercepting the circuit at the O^{ttg} operator in position P+1. On the left is the circuit with no ligand added (TFs are intercepting the recombinase from recombination), and on the right is the circuit with ligand added (TFs are detached from the operator, allowing recombination). A118 recombinase is constitutively expressed in all cases. **b** Schematic of a double-layer deletion circuit with two orthogonal attachment sites containing orthogonal O^{ttg} and O^{agg} DNA operators, enabling selective recombination: each attachment site pair with matching central conserved regions can recombine only when the ligand corresponding to their intercepting repressor is present. Repressor symbols are shown to demonstrate that these modular parts are combined in different ways to generate the data shown in **c-f**. **c-f** Assay data for different sets of repressors targeted at the circuit shown in **b**: **c** E^{+}_{HQN} and I^{+}_{KSL}, **d** E^{+}_{KSL} and R^{+}_{HQN}, **e** E^{+}_{HQN} and R^{+}_{KSL}, and **f** E^{+}_{KSL} and I^{+}_{HQN}. Source data are provided as a Source Data file. Data in **a,** and **c-f** represent the average of n = 6 biological replicates. Error bars correspond to the SEM of these measurements.

Supplementary Fig. 26

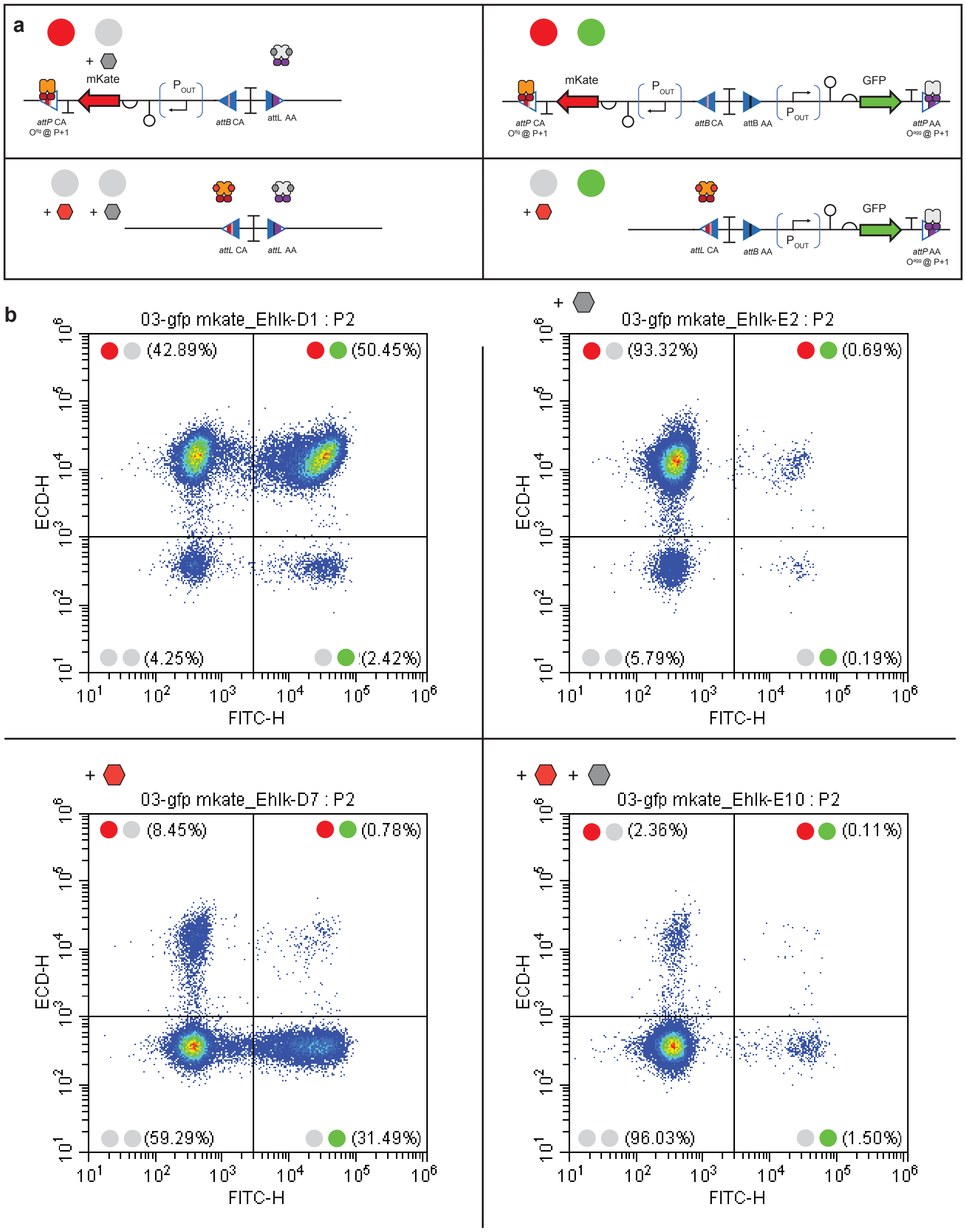

Supplementary Figure 26: Flow cytometry of a two-output circuit. a Diagram showing the genetic states corresponding to the four quadrants of each plot shown in **b**. At top right, the circuit is fully protected by the intercepting TFs E^{+}_{HQN} and I^{+}_{KSL}. E^{+}_{HQN} binds at O^{ttg} in *attP* site with CA central dinucleotide, protecting the red channel (mKate) deletion, and I^{+}_{KSL} binds at O^{agg} in *attP* site with AA central dinucleotide, protecting the green channel (sfGFP) deletion. At top left, I^{+}_{KSL} has been induced and sfGFP has been deleted. At bottom right, E^{+}_{HQN} has been induced and mKate has been deleted. At bottom left, both TFs have been induced and both fluorescent proteins have been deleted. **b** Flow cytometry data for each of the four inducer states, at top left no inducer, at top right + IPTG, at bottom left + cellobiose, and at bottom right + both IPTG and cellobiose. See Supplementary **Figure 12** for the individual performances of I^{+}_{KSL} and E^{+}_{HQN} when intercepting a single-output deletion circuit as quantified by flow cytometry. Data for the 2-OUTPUT circuit summarized in **Fig. 8b**, and Source data are provided as a Source Data file. Data represents the average of n = 6 biological replicates. Error bars correspond to the SEM of these measurements.

Supplementary Fig. 27

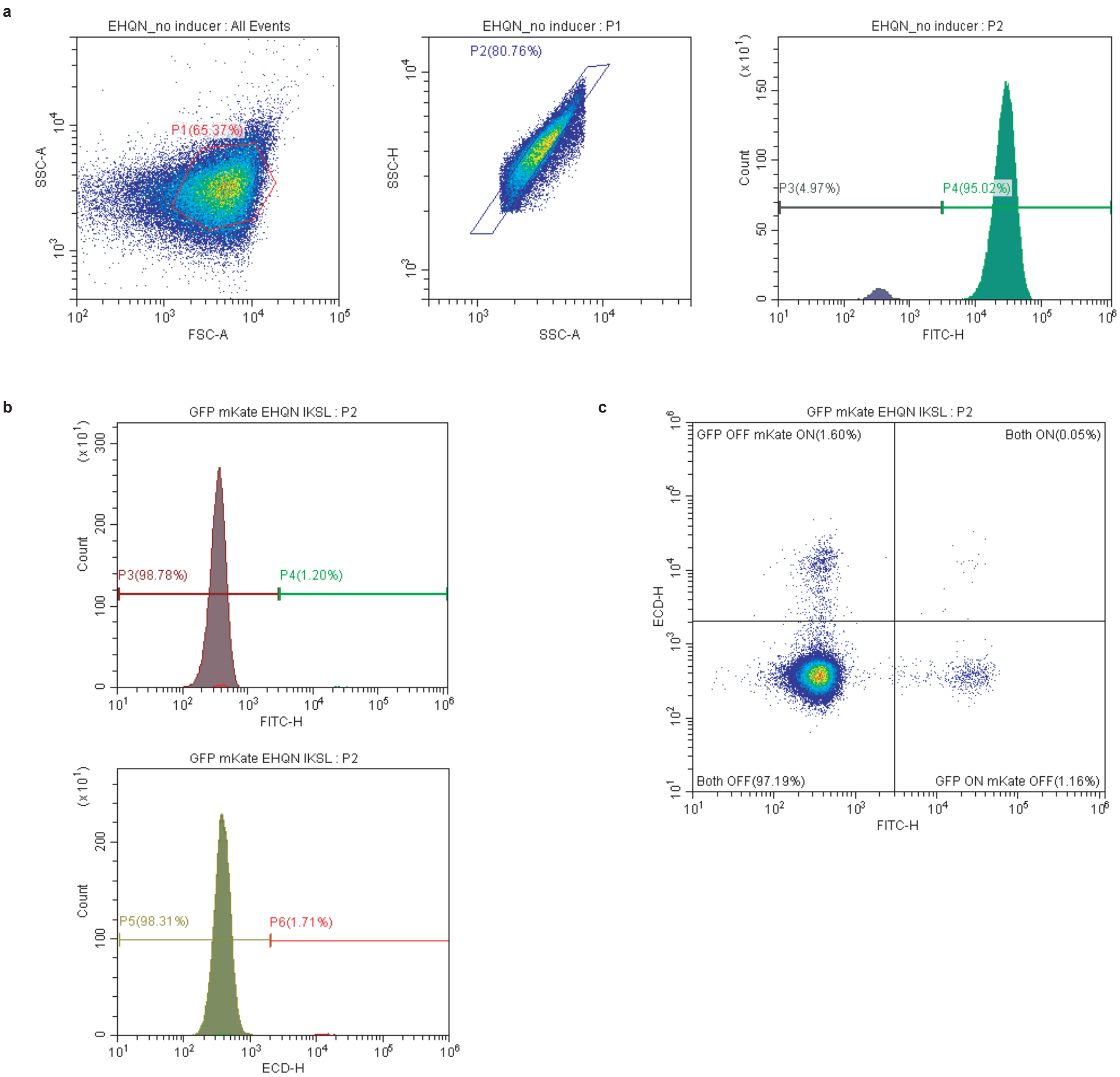

Supplementary Figure 27: Gating method for flow cytometry. The gating method used for flow cytometer analysis is shown. **a** Cells were first gated by forward scatter (FSC)-area and side scatter (SSC)-area represented as P1. Next, the gated cells were gated a second time by SSC-area and the corresponding SSC-height was represented as P2. In turn, the P2 population was gated by FITC-height value. The population having a higher than 3E3 FITC-H value were considered ON – *i.e.*, GFP expressed, and populations lower than 3E3 were considered OFF – *i.e.*, GFP not expressed. **b** The gating method used for GFP and mKate circuit is also shown. Similarly, the population higher than 3E3 FITC-H value was considered ON – *i.e.*, GFP expressed, and that lower than 3E3 was considered OFF – *i.e.*, GFP not expressed. The population higher than 2E3 ECH-H value were considered ON with mKate expressed, and that lower than 2E3 considered as OFF – *i.e.*, mKate not expressed. **c** The 2-D plot of the GFP mKate circuit is shown demonstrating the degree of the population in the OFF state. The gain for the flow cytometer settings are as follows: FSC (105), SSC (139), FITC (20), ECD (1000).

Supplementary Fig. 28

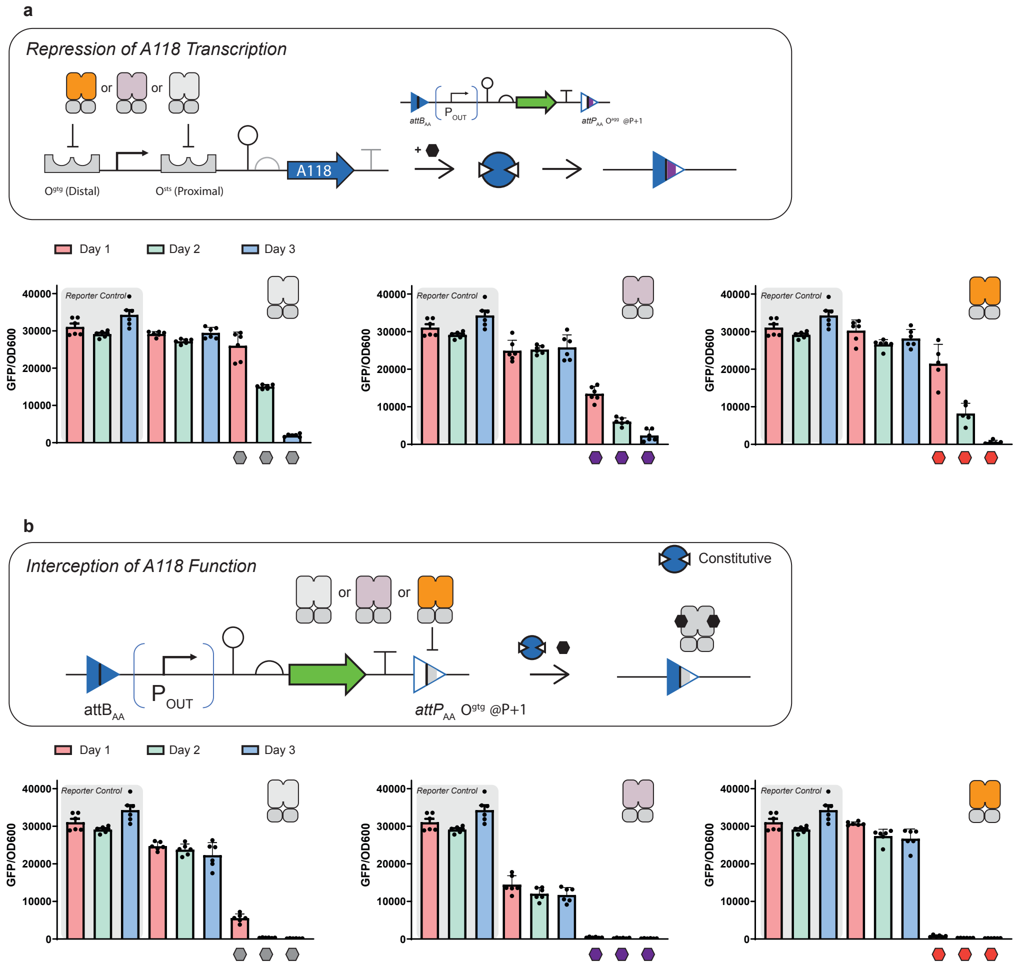

Supplementary Figure 28: Comparing type-I memory and type-II memory kinetics with constant INPUT. a Inset at top shows a schematic of the type-I memory circuit in which A118 transcription is regulated by TFs (I^{+}_{YQR}, R^{+}_{YQR}, or E^{+}_{YQR}) binding at O^{gtg} operators placed at the distal and proximal promoter positions. Inducing the repressing TF enables recombinase transcription and reporter circuit deletion. For kinetic assays, each TF is transformed with the A118-expression plasmid and reporter plasmid having O^{agg} at the P+1 position (O^{agg} is orthogonal to O^{gtg}, so the TFs are not expected to intercept at that operator). Below, kinetic assay data over three days is shown for this type-I memory circuit being repressed by (at left) I^{+}_{YQR}, (at center) R^{+}_{YQR}, and (at right) E^{+}_{YQR}. On each plot, control data for cells containing only the GFP reporter plasmid is shown at left boxed in gray. The center three bars on each plot represent the circuit with no inducer added and correspond to A118 transcription being repressed over three days. The three bars at right represent the circuit with inducer added and correspond to A118 transcription being on for three days. **b** Inset at top shows a schematic of the type-II memory circuit in which A118 function is intercepted by TFs (I^{+}_{YQR}, R^{+}_{YQR}, or E^{+}_{YQR}) binding at an O^{gtg} operator placed in the P+1 position. For kinetic assays, each TF is transformed with the A118-expression plasmid and reporter plasmid. Inducing the intercepting TF enables recombinase access to the attachment sites and reporter circuit deletion. Below, kinetic assay data over three days is shown for this type-II memory circuit being intercepted by (at left) I^{+}_{YQR}, (at center) R^{+}_{YQR}, and (at right) E^{+}_{YQR}. On each plot, control data for cells containing only the GFP reporter plasmid is shown at left boxed in gray. The center three bars on each plot represent the circuit with no inducer added and correspond to A118 function being intercepted over three days. The three bars at right represent the circuit with inducer added and correspond to A118 being unintercepted for three days. Source data are provided as a Source Data file. Data represents the average of n = 6 biological replicates. Error bars correspond to the SEM of these measurements.

Supplementary Fig. 29

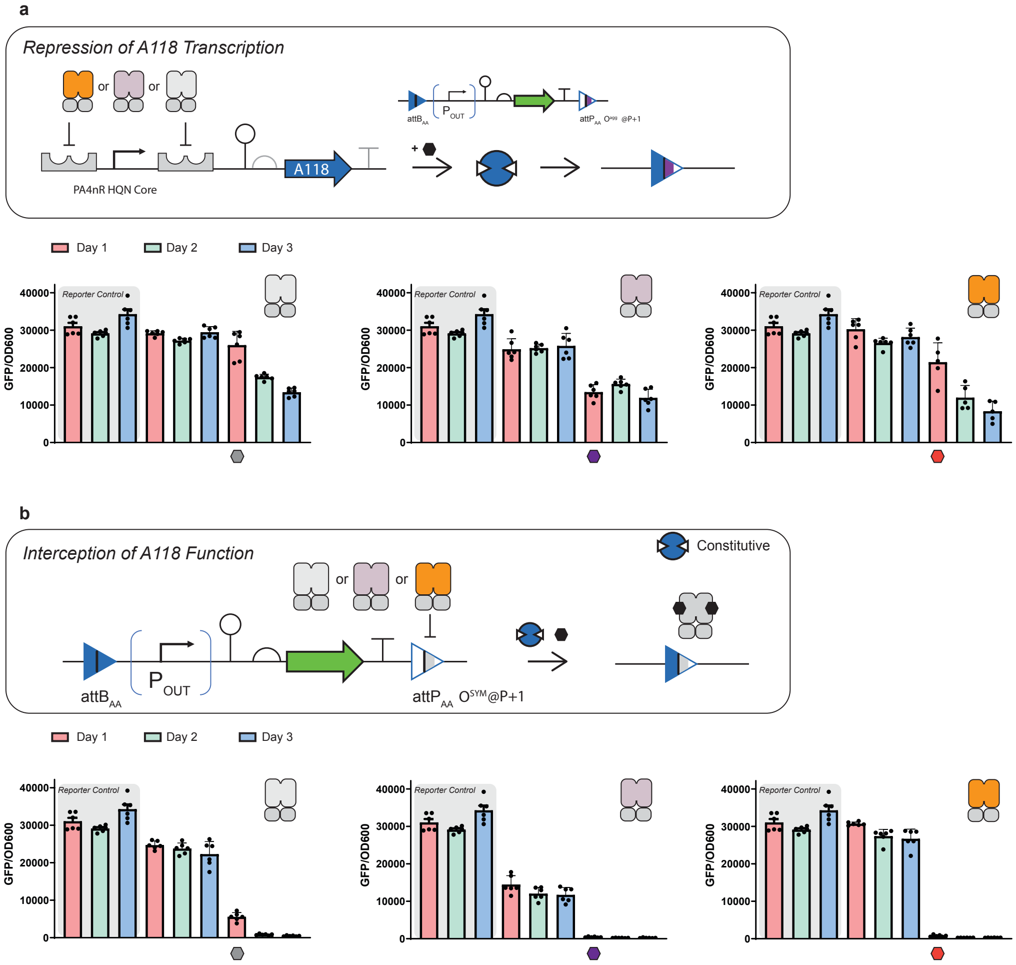

Supplementary Figure 29: Comparing type-I memory and type-II memory kinetics with transient INPUT. a-b display identical schematics to **Supplementary Figure 28**, and the constructs are subjected to equivalent experimental conditions on day 1. Following day 1, the cells are passaged as described in **Supplementary Figure 28**; however, no inducers are added, preserving the memory state written on day 1. Source data are provided as a Source Data file. Data represents the average of n = 6 biological replicates. Error bars correspond to the SEM of these measurements.

Supplementary Fig. 30

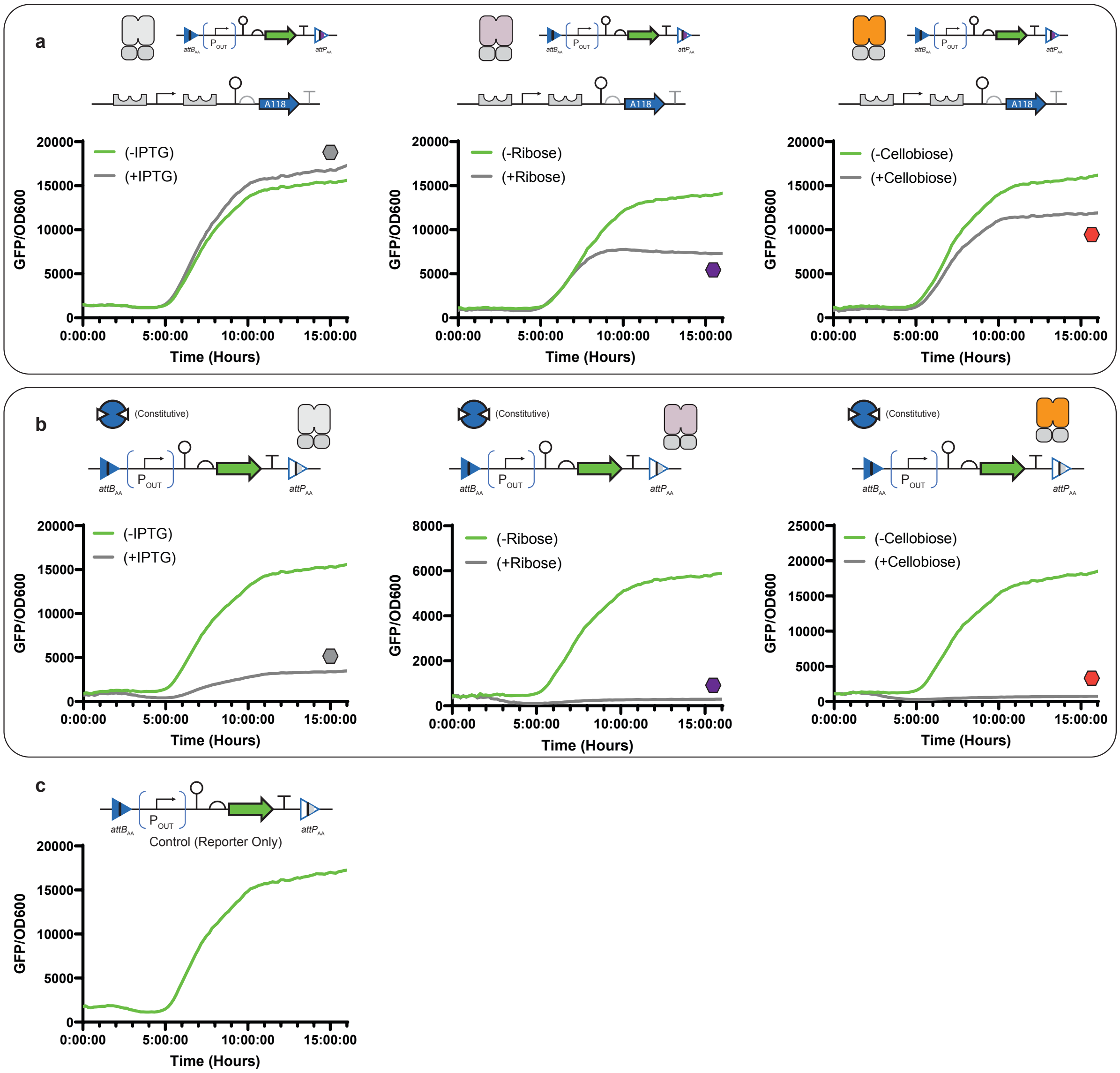

Supplementary Figure 30: Comparing type-I memory and type-II memory kinetics. Plate reader kinetic data for type-I and type-II memory. **a** At top, schematics for the type-I memory circuits used, addressed in different experiments by I^{+}_{YQR+}, R^{+}_{YQR}, or E^{+}_{YQR}. Cells were transformed with the relevant TF and an orthogonal deletion circuit (with O^{agg} at P+1) and assayed for fluorescence every 10 minutes as described in **Methods, Recombinase plate reader kinetic assays**. **b** At top, schematics for the type-II memory circuits used, addressed in different experiments by I^{+}_{YQR+}, R^{+}_{YQR}, or E^{+}_{YQR}. Cells were transformed with the relevant TF and a constitutive pSK001 A118-expression and assayed for fluorescence every 10 minutes as described in **Methods, Recombinase plate reader kinetic assays**. **c** Control experiment without the addition of recombinase. Source data are provided as a Source Data file. Data represents the average of n = 6 biological replicates. Error bars correspond to the SEM of these measurements.

Supplementary Fig. 31

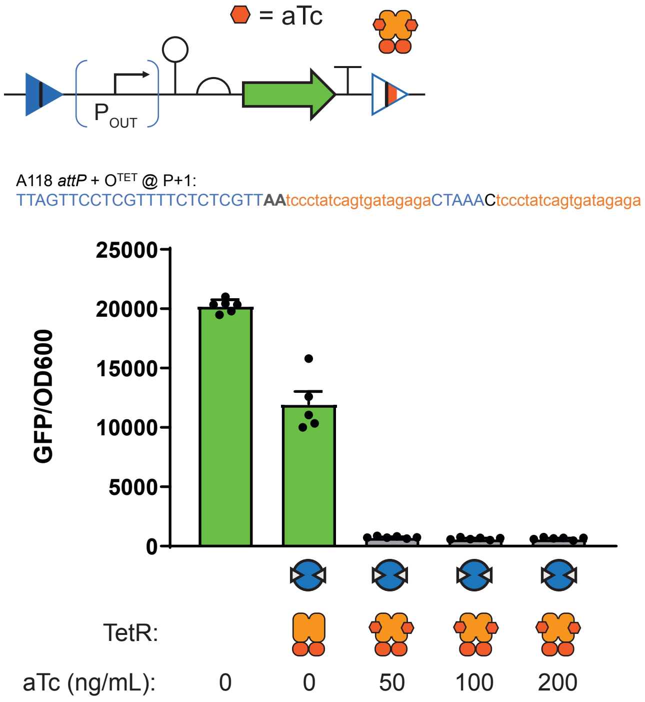

Supplementary Figure 31: Interception *via* TetR. At top, a schematic is shown for TetR intercepting a GFP deletion circuit, and the TetO operator added to A118's P+1 position is shown. At bottom, assay data for the above circuit. The leftmost bar is a control with reporter plasmid and no A118 or TetR expression. The other bars display data for all three functional plasmids being present and varying concentrations of TetR's inducer, aTc. Source data are provided as a Source Data file. Data represents the average of n = 6 biological replicates. Error bars correspond to the SEM of these measurements.

Supplementary Fig. 32

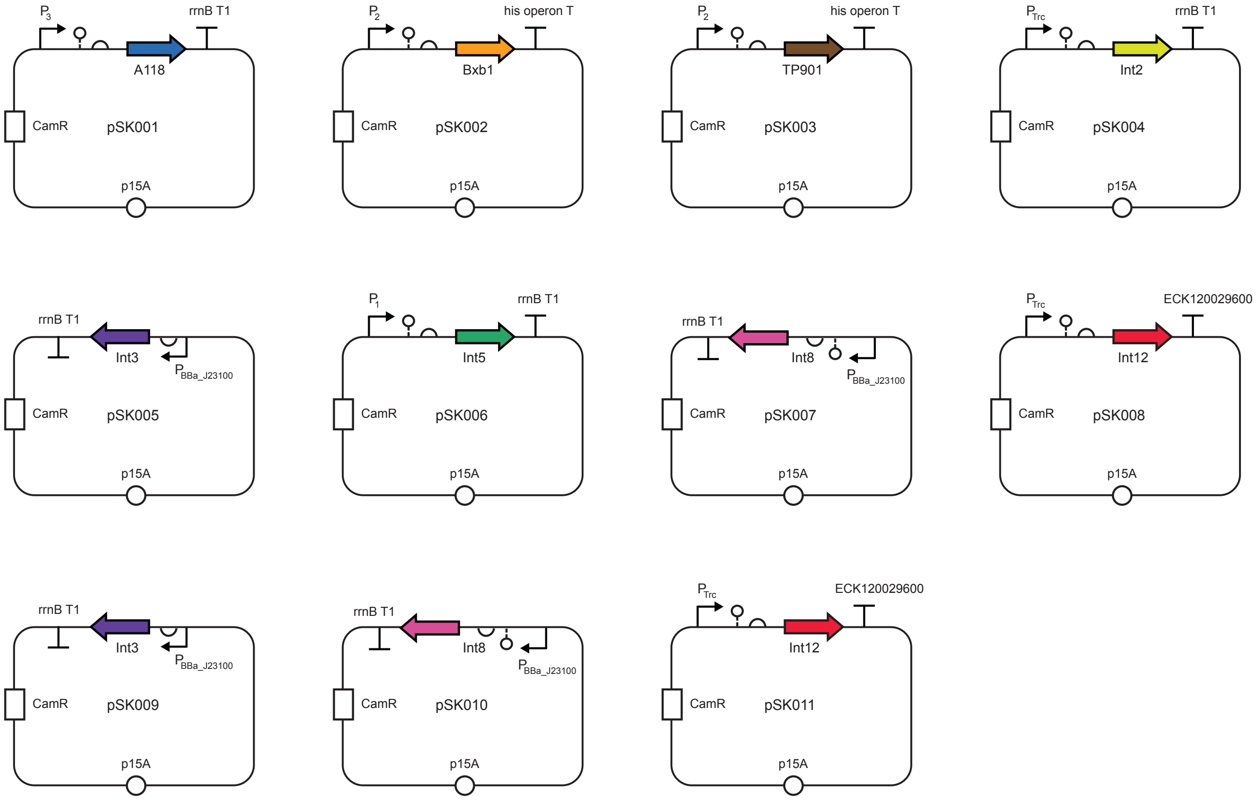

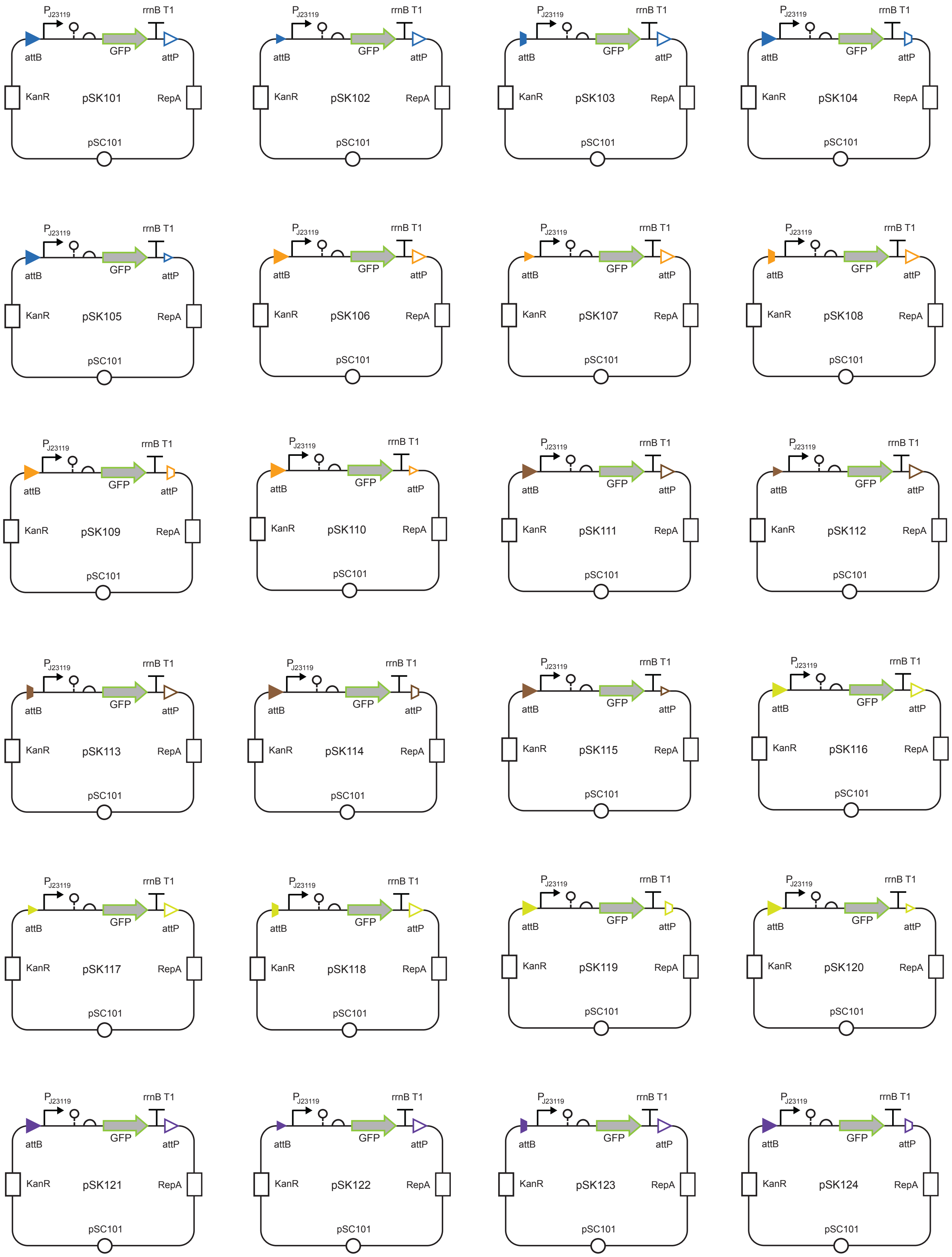

PJ23119
rrnB T1
attB
GFP
attP
KanR
pSK101
RepA
pSC101
pSK102
pSK103
pSK104
pSK105
pSK106
pSK107
pSK108
pSK109
pSK110
pSK111
pSK112
pSK113
pSK114
pSK115
pSK116
pSK117
pSK118
pSK119
pSK120
pSK121
pSK122
pSK123
pSK124

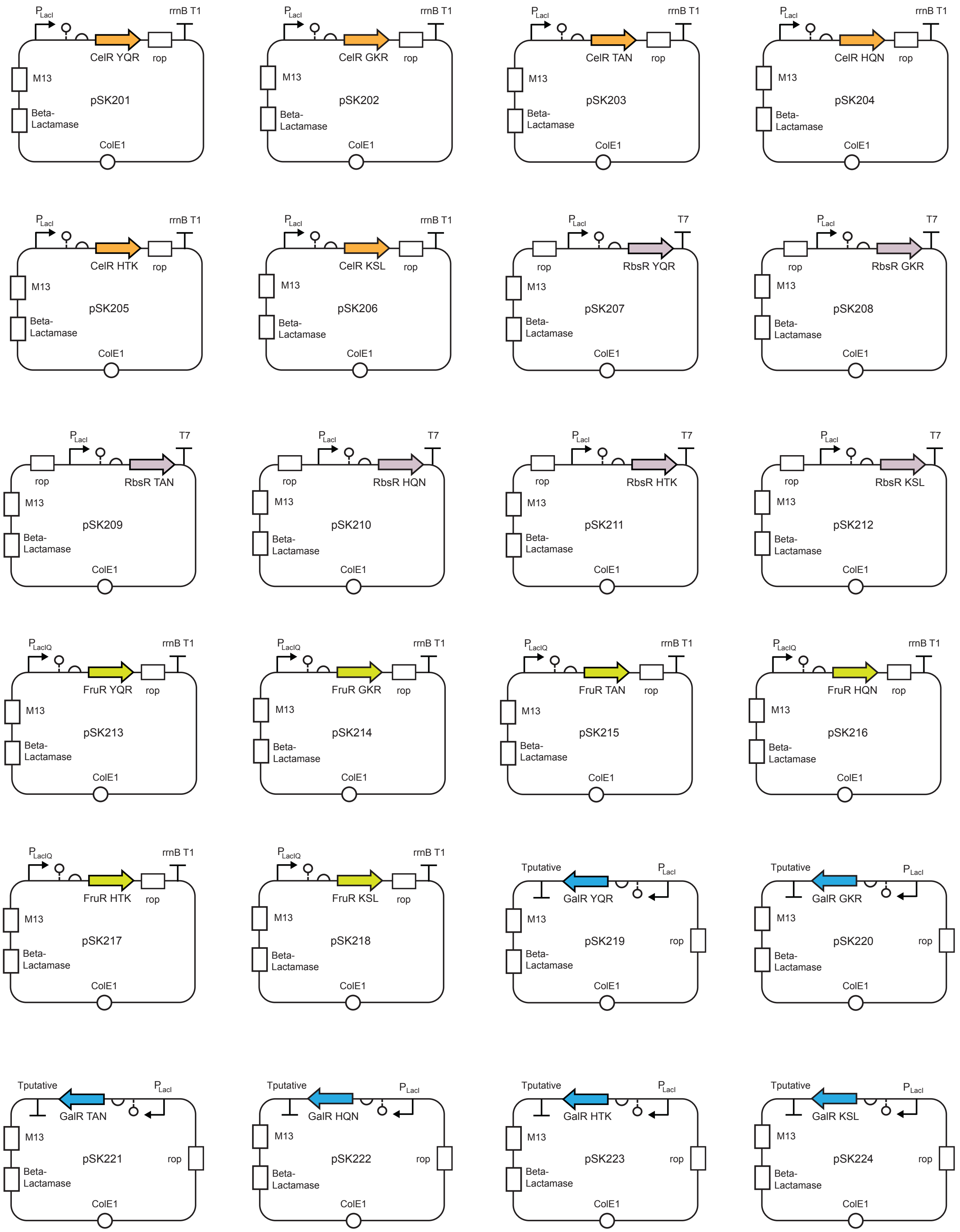
PLacI
rrnB T1
CelR YQR
rop
M13
pSK201
Beta-
Lactamase
ColE1
PLacI
rrnB T1
CelR GKR
rop
M13
pSK202
Beta-
Lactamase
ColE1
PLacI
rrnB T1
CelR TAN
rop
M13
pSK203
Beta-
Lactamase
ColE1
PLacI
rrnB T1
CelR HQN
rop
M13
pSK204
Beta-
Lactamase
ColE1
PLacI
rrnB T1
CelR HTK
rop
M13
pSK205
Beta-
Lactamase
ColE1
PLacI
rrnB T1
CelR KSL
rop
M13
pSK206
Beta-
Lactamase
ColE1
PLacI
T7
rop
RbsR YQR
M13
pSK207
Beta-
Lactamase
ColE1
PLacI
T7
rop
RbsR GKR
M13
pSK208
Beta-
Lactamase
ColE1
PLacI
T7
rop
RbsR TAN
M13
pSK209
Beta-
Lactamase
ColE1
PLacI
T7
rop
RbsR HQN
M13
pSK210
Beta-
Lactamase
ColE1
PLacI
T7
rop
RbsR HTK
M13
pSK211
Beta-
Lactamase
ColE1
PLacI
T7
rop
RbsR KSL
M13
pSK212
Beta-
Lactamase
ColE1
PLacIQ
rrnB T1
FruR YQR
rop
M13
pSK213
Beta-
Lactamase
ColE1
PLacIQ
rrnB T1
FruR GKR
rop
M13
pSK214
Beta-
Lactamase
ColE1
PLacIQ
rrnB T1
FruR TAN
rop
M13
pSK215
Beta-
Lactamase
ColE1
PLacIQ
rrnB T1
FruR HQN
rop
M13
pSK216
Beta-
Lactamase
ColE1
PLacIQ
rrnB T1
FruR HTK
rop
M13
pSK217
Beta-
Lactamase
ColE1
PLacIQ
rrnB T1
FruR KSL
rop
M13
pSK218
Beta-
Lactamase
ColE1
Tputative
PLacI
GalR YQR
M13
pSK219
rop
Beta-
Lactamase
ColE1
Tputative
PLacI
GalR GKR
M13
pSK220
rop
Beta-
Lactamase
ColE1
Tputative
PLacI
GalR TAN
M13
pSK221
rop
Beta-
Lactamase
ColE1
Tputative
PLacI
GalR HQN
M13
pSK222
rop
Beta-
Lactamase
ColE1
Tputative
PLacI
GalR HTK
M13
pSK223
rop
Beta-
Lactamase
ColE1
Tputative
PLacI
GalR KSL
M13
pSK224
rop
Beta-
Lactamase
ColE1

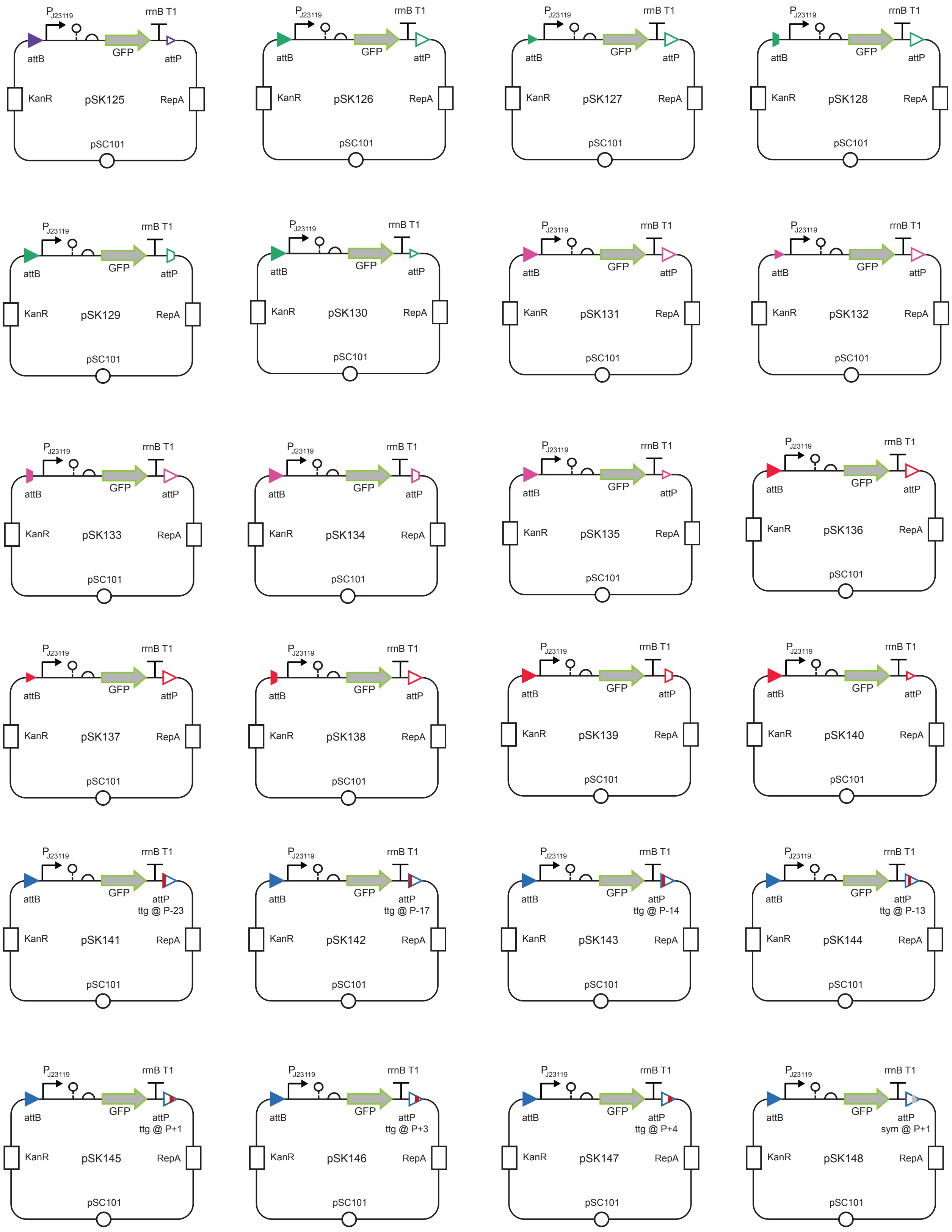

PJ23119
rrnB T1
attB
GFP
attP
KanR
pSK125
RepA
pSC101
PJ23119
rrnB T1
attB
GFP
attP
KanR
pSK126
RepA
pSC101
PJ23119
rrnB T1
attB
GFP
attP
KanR
pSK127
RepA
pSC101
PJ23119
rrnB T1
attB
GFP
attP
KanR
pSK128
RepA
pSC101
PJ23119
rrnB T1
attB
GFP
attP
KanR
pSK129
RepA
pSC101
PJ23119
rrnB T1
attB
GFP
attP
KanR
pSK130
RepA
pSC101
PJ23119
rrnB T1
attB
GFP
attP
KanR
pSK131
RepA
pSC101
PJ23119
rrnB T1
attB
GFP
attP
KanR
pSK132
RepA
pSC101
PJ23119
rrnB T1
attB
GFP
attP
KanR
pSK133
RepA
pSC101
PJ23119
rrnB T1
attB
GFP
attP
KanR
pSK134
RepA
pSC101
PJ23119
rrnB T1
attB
GFP
attP
KanR
pSK135
RepA
pSC101
PJ23119
rrnB T1
attB
GFP
attP
KanR
pSK136
RepA
pSC101
PJ23119
rrnB T1
attB
GFP
attP
KanR
pSK137
RepA
pSC101
PJ23119
rrnB T1
attB
GFP
attP
KanR
pSK138
RepA
pSC101
PJ23119
rrnB T1
attB
GFP
attP
KanR
pSK139
RepA
pSC101
PJ23119
rrnB T1
attB
GFP
attP
KanR
pSK140
RepA
pSC101
PJ23119
rrnB T1
attB
GFP
attP
ttg @ P-23
KanR
pSK141
RepA
pSC101
PJ23119
rrnB T1
attB
GFP
attP
ttg @ P-17
KanR
pSK142
RepA
pSC101
PJ23119
rrnB T1
attB
GFP
attP
ttg @ P-14
KanR
pSK143
RepA
pSC101
PJ23119
rrnB T1
attB
GFP
attP
ttg @ P-13
KanR
pSK144
RepA
pSC101
PJ23119
rrnB T1
attB
GFP
attP
ttg @ P+1
KanR
pSK145
RepA
pSC101
PJ23119
rrnB T1
attB
GFP
attP
ttg @ P+3
KanR
pSK146
RepA
pSC101
PJ23119
rrnB T1
attB
GFP
attP
ttg @ P+4
KanR
pSK147
RepA
pSC101
PJ23119
rrnB T1
attB
GFP
attP
sym @ P+1
KanR
pSK148
RepA
pSC101

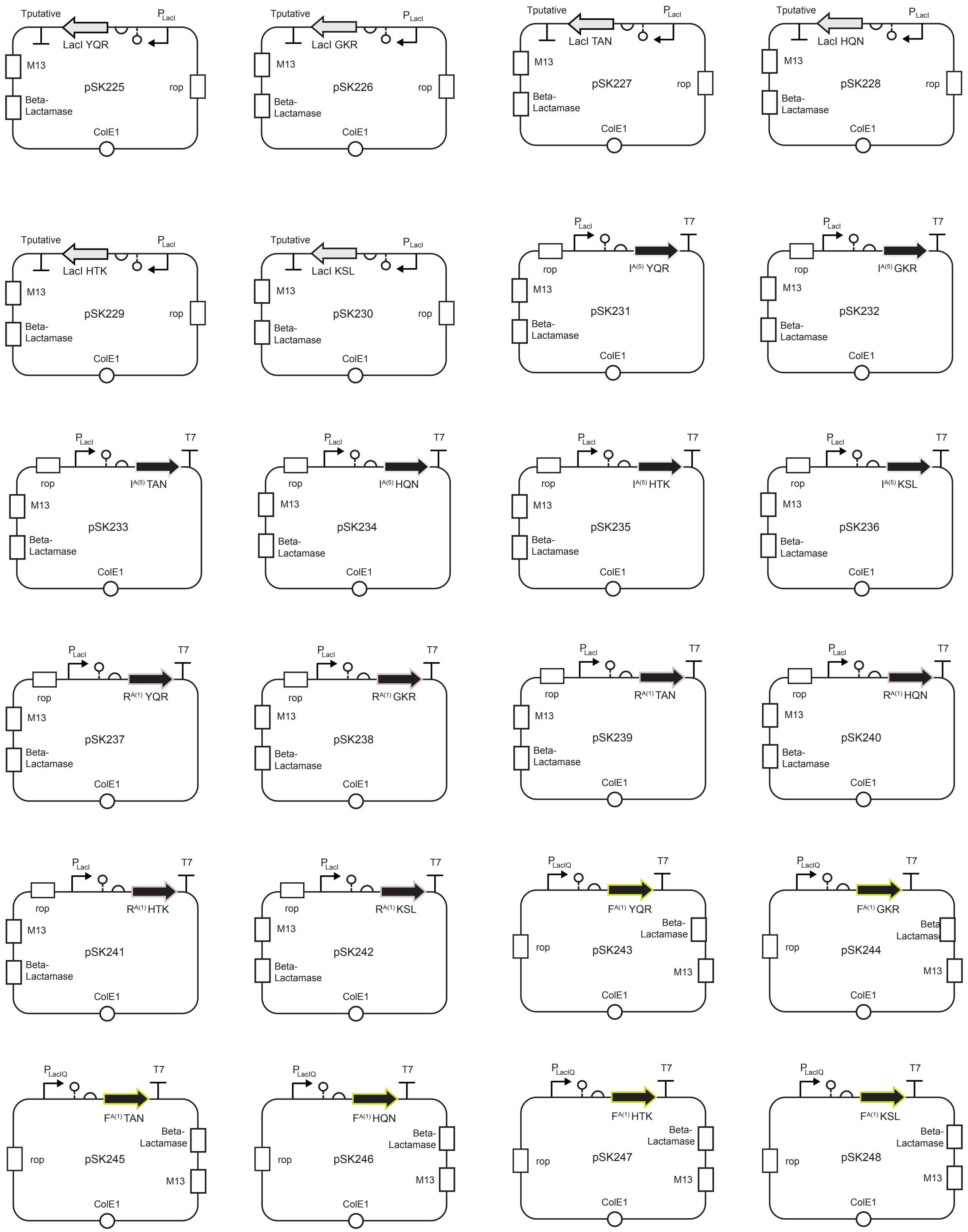
Tputative
P_{LacI}
LacI YQR
M13
pSK225
rop
Beta-Lactamase
ColE1
Tputative
P_{LacI}
LacI GKR
M13
pSK226
rop
Beta-Lactamase
ColE1
Tputative
P_{LacI}
LacI TAN
M13
pSK227
rop
Beta-Lactamase
ColE1
Tputative
P_{LacI}
LacI HQN
M13
pSK228
rop
Beta-Lactamase
ColE1
Tputative
P_{LacI}
LacI HTK
M13
pSK229
rop
Beta-Lactamase
ColE1
Tputative
P_{LacI}
LacI KSL
M13
pSK230
rop
Beta-Lactamase
ColE1
P_{LacI}
T7
rop
$I^{A(5)}$ YQR
M13
pSK231
Beta-Lactamase
ColE1
P_{LacI}
T7
rop
$I^{A(5)}$ GKR
M13
pSK232
Beta-Lactamase
ColE1
P_{LacI}
T7
rop
$I^{A(5)}$ TAN
M13
pSK233
Beta-Lactamase
ColE1
P_{LacI}
T7
rop
$I^{A(5)}$ HQN
M13
pSK234
Beta-Lactamase
ColE1
P_{LacI}
T7
rop
$I^{A(5)}$ HTK
M13
pSK235
Beta-Lactamase
ColE1
P_{LacI}
T7
rop
$I^{A(5)}$ KSL
M13
pSK236
Beta-Lactamase
ColE1
P_{LacI}
T7
rop
$R^{A(1)}$ YQR
M13
pSK237
Beta-Lactamase
ColE1
P_{LacI}
T7
rop
$R^{A(1)}$ GKR
M13
pSK238
Beta-Lactamase
ColE1
P_{LacI}
T7
rop
$R^{A(1)}$ TAN
M13
pSK239
Beta-Lactamase
ColE1
P_{LacI}
T7
rop
$R^{A(1)}$ HQN
M13
pSK240
Beta-Lactamase
ColE1
P_{LacI}
T7
rop
$R^{A(1)}$ HTK
M13
pSK241
Beta-Lactamase
ColE1
P_{LacI}
T7
rop
$R^{A(1)}$ KSL
M13
pSK242
Beta-Lactamase
ColE1
P_{LacIQ}
T7
$F^{A(1)}$ YQR
Beta-Lactamase
rop
pSK243
M13
ColE1
P_{LacIQ}
T7
$F^{A(1)}$ GKR
Beta-Lactamase
rop
pSK244
M13
ColE1
P_{LacIQ}
T7
$F^{A(1)}$ TAN
Beta-Lactamase
rop
pSK245
M13
ColE1
P_{LacIQ}
T7
$F^{A(1)}$ HQN
Beta-Lactamase
rop
pSK246
M13
ColE1
P_{LacIQ}
T7
$F^{A(1)}$ HTK
Beta-Lactamase
rop
pSK247
M13
ColE1
P_{LacIQ}
T7
$F^{A(1)}$ KSL
Beta-Lactamase
rop
pSK248
M13
ColE1

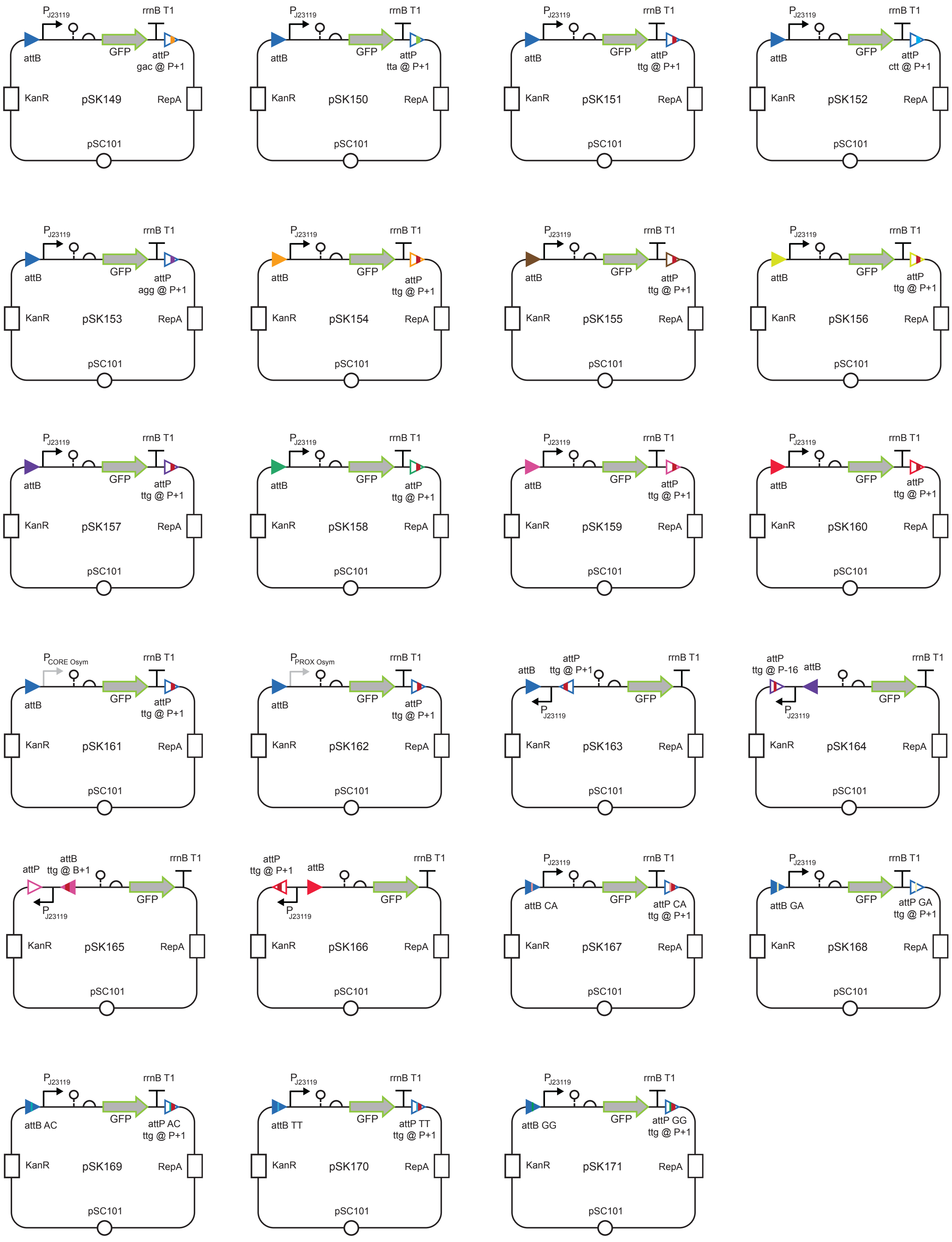

pSK149
P_{J23119}
rrnB T1
attB
GFP
attP
gac @ P+1
KanR
RepA
pSC101
pSK150
P_{J23119}
rrnB T1
attB
GFP
attP
tta @ P+1
KanR
RepA
pSC101
pSK151
P_{J23119}
rrnB T1
attB
GFP
attP
ttg @ P+1
KanR
RepA
pSC101
pSK152
P_{J23119}
rrnB T1
attB
GFP
attP
ctt @ P+1
KanR
RepA
pSC101
pSK153
P_{J23119}
rrnB T1
attB
GFP
attP
agg @ P+1
KanR
RepA
pSC101
pSK154
P_{J23119}
rrnB T1
attB
GFP
attP
ttg @ P+1
KanR
RepA
pSC101
pSK155
P_{J23119}
rrnB T1
attB
GFP
attP
ttg @ P+1
KanR
RepA
pSC101
pSK156
P_{J23119}
rrnB T1
attB
GFP
attP
ttg @ P+1
KanR
RepA
pSC101
pSK157
P_{J23119}
rrnB T1
attB
GFP
attP
ttg @ P+1
KanR
RepA
pSC101
pSK158
P_{J23119}
rrnB T1
attB
GFP
attP
ttg @ P+1
KanR
RepA
pSC101
pSK159
P_{J23119}
rrnB T1
attB
GFP
attP
ttg @ P+1
KanR
RepA
pSC101
pSK160
P_{J23119}
rrnB T1
attB
GFP
attP
ttg @ P+1
KanR
RepA
pSC101
pSK161
$P_{CORE\ Osym}$
rrnB T1
attB
GFP
attP
ttg @ P+1
KanR
RepA
pSC101
pSK162
$P_{PROX\ Osym}$
rrnB T1
attB
GFP
attP
ttg @ P+1
KanR
RepA
pSC101
pSK163
attB
attP
ttg @ P+1
P_{J23119}
GFP
rrnB T1
KanR
RepA
pSC101
pSK164
attP
ttg @ P-16
attB
P_{J23119}
GFP
rrnB T1
KanR
RepA
pSC101
pSK165
attP
attB
ttg @ B+1
P_{J23119}
GFP
rrnB T1
KanR
RepA
pSC101
pSK166
attP
ttg @ P+1
attB
P_{J23119}
GFP
rrnB T1
KanR
RepA
pSC101
pSK167
P_{J23119}
rrnB T1
attB CA
GFP
attP CA
ttg @ P+1
KanR
RepA
pSC101
pSK168
P_{J23119}
rrnB T1
attB GA
GFP
attP GA
ttg @ P+1
KanR
RepA
pSC101
pSK169
P_{J23119}
rrnB T1
attB AC
GFP
attP AC
ttg @ P+1
KanR
RepA
pSC101
pSK170
P_{J23119}
rrnB T1
attB TT
GFP
attP TT
ttg @ P+1
KanR
RepA
pSC101
pSK171
P_{J23119}
rrnB T1
attB GG
GFP
attP GG
ttg @ P+1
KanR
RepA
pSC101

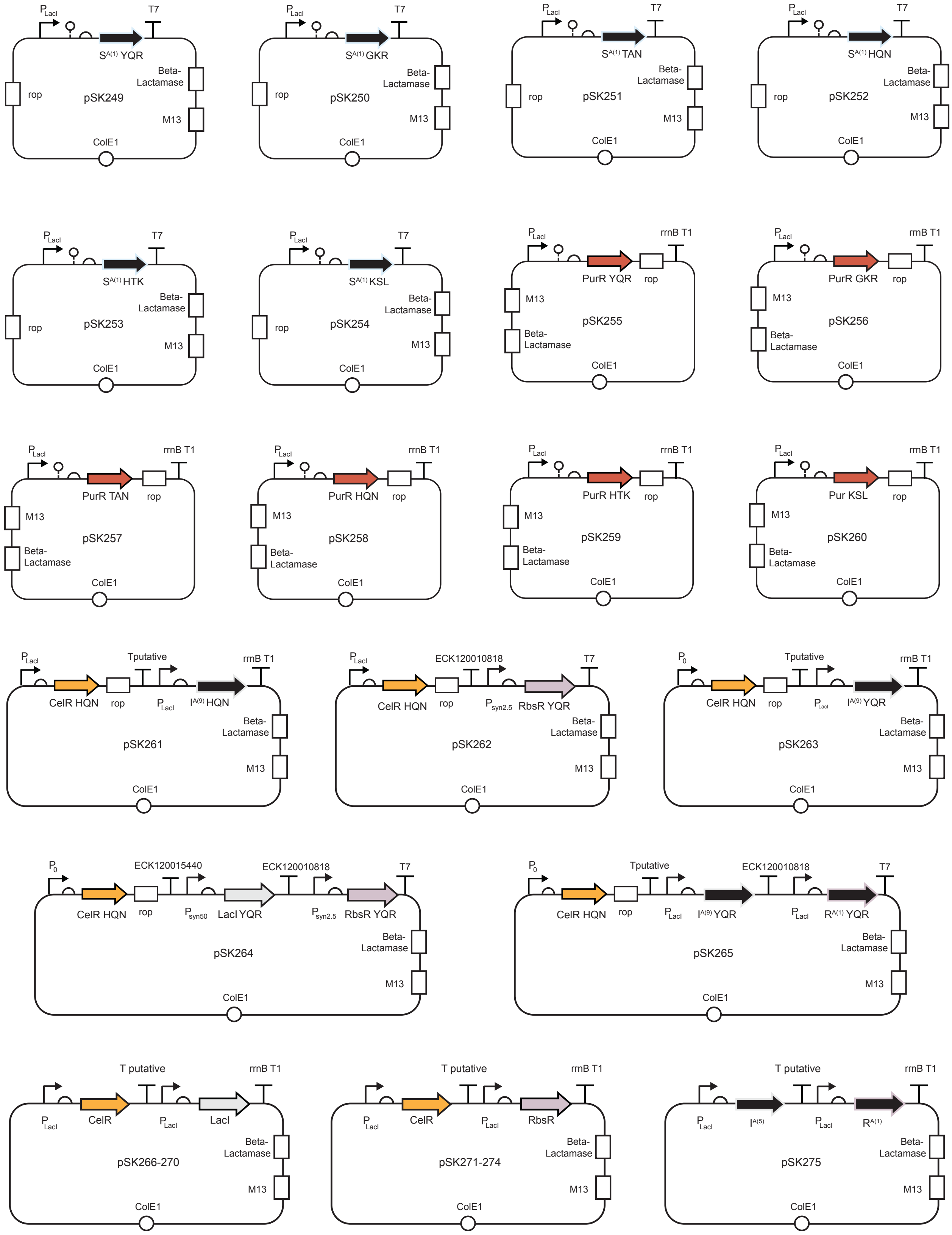
PLacI
T7
SA(1) YQR
Beta-Lactamase
rop
pSK249
M13
ColE1
PLacI
T7
SA(1) GKR
Beta-Lactamase
rop
pSK250
M13
ColE1
PLacI
T7
SA(1) TAN
Beta-Lactamase
rop
pSK251
M13
ColE1
PLacI
T7
SA(1) HQN
Beta-Lactamase
rop
pSK252
M13
ColE1
PLacI
T7
SA(1) HTK
Beta-Lactamase
rop
pSK253
M13
ColE1
PLacI
T7
SA(1) KSL
Beta-Lactamase
rop
pSK254
M13
ColE1
PLacI
rrnB T1
PurR YQR
rop
M13
pSK255
Beta-Lactamase
ColE1
PLacI
rrnB T1
PurR GKR
rop
M13
pSK256
Beta-Lactamase
ColE1
PLacI
rrnB T1
PurR TAN
rop
M13
pSK257
Beta-Lactamase
ColE1
PLacI
rrnB T1
PurR HQN
rop
M13
pSK258
Beta-Lactamase
ColE1
PLacI
rrnB T1
PurR HTK
rop
M13
pSK259
Beta-Lactamase
ColE1
PLacI
rrnB T1
Pur KSL
rop
M13
pSK260
Beta-Lactamase
ColE1
PLacI
Tputative
rrnB T1
CelR HQN
rop
PLacI
IA(9) HQN
Beta-Lactamase
pSK261
M13
ColE1
PLacI
ECK120010818
T7
CelR HQN
rop
Psyn2.5
RbsR YQR
Beta-Lactamase
pSK262
M13
ColE1
P0
Tputative
rrnB T1
CelR HQN
rop
PLacI
IA(9) YQR
Beta-Lactamase
pSK263
M13
ColE1
P0
ECK120015440
ECK120010818
T7
CelR HQN
rop
Psyn50
LacI YQR
Psyn2.5
RbsR YQR
Beta-Lactamase
pSK264
M13
ColE1
P0
Tputative
ECK120010818
T7
CelR HQN
rop
PLacI
IA(9) YQR
PLacI
RA(1) YQR
Beta-Lactamase
pSK265
M13
ColE1
T putative
rrnB T1
PLacI
CelR
PLacI
LacI
Beta-Lactamase
pSK266-270
M13
ColE1
T putative
rrnB T1
PLacI
CelR
PLacI
RbsR
Beta-Lactamase
pSK271-274
M13
ColE1
T putative
rrnB T1
PLacI
IA(5)
PLacI
RA(1)
Beta-Lactamase
pSK275
M13
ColE1

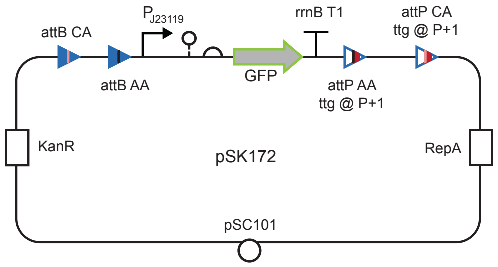

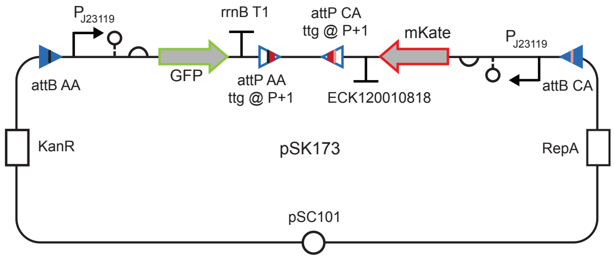

Supplementary Figure 32: Relevant plasmid maps used in this study.

Supplementary Fig. 4 (uncropped gels)

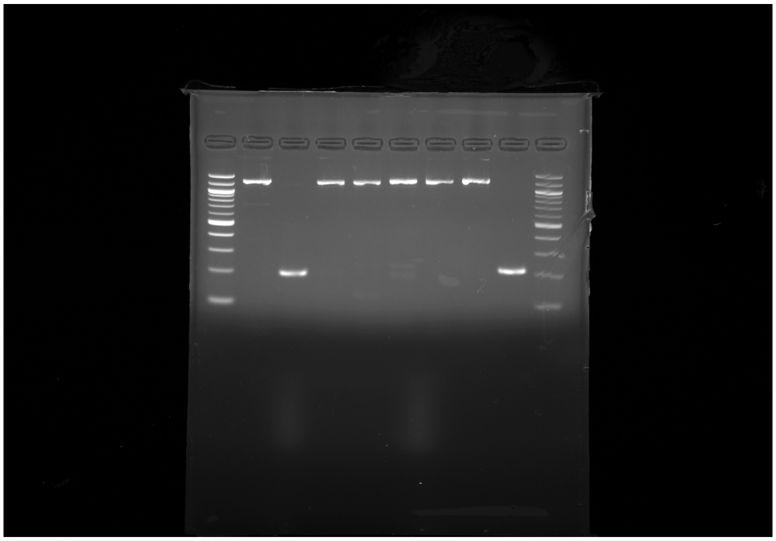

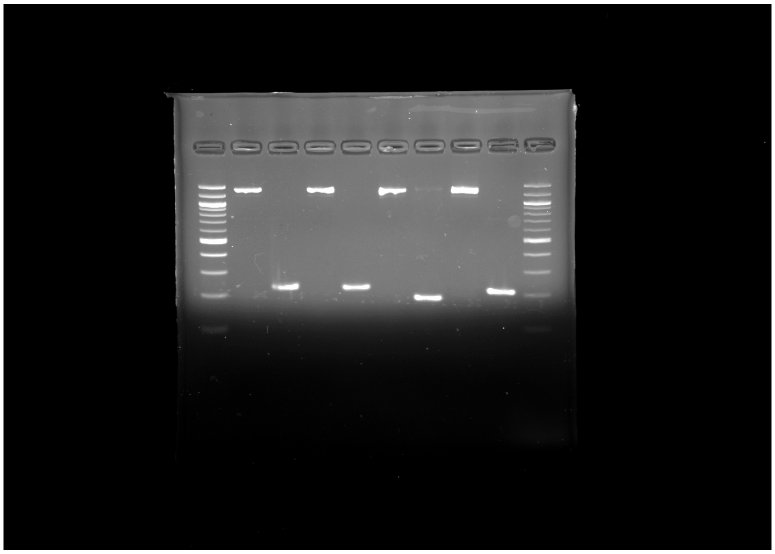

Supplementary Figure 4: Qualitative genotype of 8 recombinases paired with cognate interception deletion circuits. uncropped gels – details and analysis given on page 7 of this SI document.

Supplementary Table 1

**Transcription Factor**	**Day 1 Dynamic Range** $\frac{Fl.for - Inducer}{Fl.for + Inducer}$	**Day 2 Dynamic Range** $\frac{Fl.for - Inducer}{Fl.for + Inducer}$	**Day 3 Dynamic Range** $\frac{Fl.for - Inducer}{Fl.for + Inducer}$
E^{+}_{HQN}	20.63	87.93	114.83
E^{+}_{KSL}	14.73	49.84	71.51
E^{+}_{YQR}	20.91	85.96	141.52
I^{+}_{HQN}	15.43	35.23	57.65
I^{+}_{KSL}	5.44	6.04	7.42
I^{+}_{YQR}	9.26	21.14	29.77

Supplementary Table 1: Maintenance of interception of Loss of Function circuits over three days. The longitudinal stability of Interception in loss of function circuits over three days is shown. The intercepted state was maintained over three days, as shown as Dynamic Ranges. Six transcription factors (E^{+}_{HQN}, E^{+}_{KSL}, E^{+}_{YQR}, I^{+}_{HQN}, I^{+}_{KSL}, and I^{+}_{YQR}) were tested over three days. The Dynamic Range was calculated as the ratio of fluorescence value for – inducer to fluorescence value for + inducer, representing the ratio of intercepted state to the recombined state. Over the period, the dynamic range increased, indicating that the stability of the interception also increased. The dynamic range value represents the average of n = 6 biological replicates. Source data are provided as a Source Data File.

1. Smith, M.C.A., Till, R. & Smith, M.C.M. Switching the polarity of a bacteriophage integration system. *Molecular Microbiology* **51**, 1719-1728 (2004).
2. Milner, P.T. *et al.* Performance Prediction of Fundamental Transcriptional Programs. *ACS Synth Biol* (2023).
