# Supplementary material for: Next generation synthetic memory via intercepting recombinase function
Source: Nat Commun. 2023 Aug 29;14:5255. doi: 10.1038/s41467-023-41043-w (PMC10465543; doi:10.1038/s41467-023-41043-w)
Supplement: Supplementary file 2 — Reporting Summary [file 41467_2023_41043_MOESM2_ESM.pdf]

nature portfolio

Corresponding author(s): Corey J. Wilson

Last updated by author(s): Aug 9, 2023

Reporting Summary

Nature Portfolio wishes to improve the reproducibility of the work that we publish. This form provides structure for consistency and transparency in reporting. For further information on Nature Portfolio policies, see our Editorial Policies and the Editorial Policy Checklist.

Statistics

For all statistical analyses, confirm that the following items are present in the figure legend, table legend, main text, or Methods section.

n/a	Confirmed	
☐	☒	The exact sample size (*n*) for each experimental group/condition, given as a discrete number and unit of measurement
☐	☒	A statement on whether measurements were taken from distinct samples or whether the same sample was measured repeatedly
☐	☒	The statistical test(s) used AND whether they are one- or two-sided<br>*Only common tests should be described solely by name; describe more complex techniques in the Methods section.*
☒	☐	A description of all covariates tested
☒	☐	A description of any assumptions or corrections, such as tests of normality and adjustment for multiple comparisons
☐	☒	A full description of the statistical parameters including central tendency (e.g. means) or other basic estimates (e.g. regression coefficient) AND variation (e.g. standard deviation) or associated estimates of uncertainty (e.g. confidence intervals)
☒	☐	For null hypothesis testing, the test statistic (e.g. *F*, *t*, *r*) with confidence intervals, effect sizes, degrees of freedom and *P* value noted<br>*Give P values as exact values whenever suitable.*
☒	☐	For Bayesian analysis, information on the choice of priors and Markov chain Monte Carlo settings
☒	☐	For hierarchical and complex designs, identification of the appropriate level for tests and full reporting of outcomes
☒	☐	Estimates of effect sizes (e.g. Cohen's *d*, Pearson's *r*), indicating how they were calculated

Our web collection on statistics for biologists contains articles on many of the points above.

Software and code

Policy information about availability of computer code

Data collection	For microwell plate assay, Molecular Devices SoftMax Pro (version 7.0.3) was used to collect plate reader data. For flow cytometry, CytExpert 2.5 was used for cell population data.
Data analysis	For data analysis, Microsoft Excel (2021), Graphpad Prism (version 9.3.1) were used. ApE Plasmid Editor (version 3.1.3) and SnapGene Viewer (version 5.0.7) were used to visualize and modify the plasmid map. Random DNA Sequence Generator (http://faculty.ucr.edu/~mmaduro/random.htm) was used to generate the random DNA sequence for deletion test. RBS Library Calculator (version 2.0, https://salislab.net/software), NEBuilder Assembly Tool (version 2.8.2, https://nebuilder.neb.com/#!/), and IDT Oligo Analyzer (version 3.1, https://www.idtdna.com/calc/analyzer) were used to design and build the primers for DNA constructs.

For manuscripts utilizing custom algorithms or software that are central to the research but not yet described in published literature, software must be made available to editors and reviewers. We strongly encourage code deposition in a community repository (e.g. GitHub). See the Nature Portfolio guidelines for submitting code & software for further information.

Data

Policy information about availability of data

All manuscripts must include a data availability statement. This statement should provide the following information, where applicable:

- Accession codes, unique identifiers, or web links for publicly available datasets
- A description of any restrictions on data availability
- For clinical datasets or third party data, please ensure that the statement adheres to our policy

The authors declare that all data supporting the findings of this study are available within the paper and its Supplementary Information. The analyzed data generated in this study are provided in the Supplementary Information. Source Data are provided with this paper. The plasmid maps used in this study have been deposited in the GenBank (NIH) database under accession code pSK001 - pSK012 (OR187764 - OR187775), pSK101 - pSK173 (OR187776 - OR187811), pSK201 - pSK275 (OR187812 - OR187829). Additional questions will be answered by the corresponding author on reasonable request.

Research involving human participants, their data, or biological material

Policy information about studies with human participants or human data. See also policy information about sex, gender (identity/presentation), and sexual orientation and race, ethnicity and racism.

Reporting on sex and gender	This research did not involve human participants, their data, or their biological materials.
Reporting on race, ethnicity, or other socially relevant groupings	This research did not involve human participants, their data, or their biological materials.
Population characteristics	This research did not involve human participants, their data, or their biological materials.
Recruitment	This research did not involve human participants, their data, or their biological materials.
Ethics oversight	This research did not involve human participants, their data, or their biological materials.

Note that full information on the approval of the study protocol must also be provided in the manuscript.

Field-specific reporting

Please select the one below that is the best fit for your research. If you are not sure, read the appropriate sections before making your selection.

- [x] Life sciences
- [ ] Behavioural & social sciences
- [ ] Ecological, evolutionary & environmental sciences

For a reference copy of the document with all sections, see nature.com/documents/nr-reporting-summary-flat.pdf

Life sciences study design

All studies must disclose on these points even when the disclosure is negative.

Sample size	All of the sample size performed in each experiment is indicated in the figure legends. In general, sample size of n=6 was chosen for fluorescence assays based on previous works published in Nat. comm. (Rondon et. al., 2019), (Groseclose et. al., 2020), (Huang et. al., 2022). For the assay, n=6 biological replicates (individual colonies) were performed per day, with certain assays repeated on a second and third day to provide kinetic data.
Data exclusions	No data has been excluded from analyses. All data collected is shown on plots and included in the Source Data file.
Replication	The following experiments were repeated on 2 separate days with n=6 biological replicates on each day: interception of A118 recombinase at Ottg in the P+1 position, at Ogtg in the P+1 position, and at Oagg in the P+1 position by the RCDs LacI, RbsR, and CelR with corresponding DBDs (see Fig. 2). This was done to confirm the reproducibility of the assay and logic gates. All experiments were faithfully reproduced within 1 standard deviation of the original. Data for all other experiments were derived from independent biological replicates taken on the same day.
Randomization	Biological replicates were randomized by picking single colonies from the LB agar plates after transformation. Each single colonies represents a single biological replicate, so sample size n=6 represents biological sixplicate. All experiments were conducted n=6 by picking 6 randomized colonies for assay.
Blinding	Blinding was not relevant to this study because knowledge of genotype and plasmid sequence was required for experiments to be performed under the correct conditions.

Reporting for specific materials, systems and methods

We require information from authors about some types of materials, experimental systems and methods used in many studies. Here, indicate whether each material, system or method listed is relevant to your study. If you are not sure if a list item applies to your research, read the appropriate section before selecting a response.

Materials & experimental systems

n/a	Involved in the study
☒	☐ Antibodies
☒	☐ Eukaryotic cell lines
☒	☐ Palaeontology and archaeology
☒	☐ Animals and other organisms
☒	☐ Clinical data
☒	☐ Dual use research of concern
☒	☐ Plants

Methods

n/a	Involved in the study
☒	☐ ChIP-seq
☐	☒ Flow cytometry
☒	☐ MRI-based neuroimaging

Flow Cytometry

Plots

Confirm that:

☒ The axis labels state the marker and fluorochrome used (e.g. CD4-FITC).

☒ The axis scales are clearly visible. Include numbers along axes only for bottom left plot of group (a 'group' is an analysis of identical markers).

☒ All plots are contour plots with outliers or pseudocolor plots.

☒ A numerical value for number of cells or percentage (with statistics) is provided.

Methodology

Sample preparation	E. coli 3.32 cells were grown as described in "Methods, Microwell plate assay", then passaged for an additional 24 hours with no inducer as described in "Methods, Recombinase three-day kinetic assays". Cells were then diluted 1:19 into phosphate-buffered saline (PBS) solution with 2mg/ml kanamycin and incubated for at least 1 hour at room temperature to inhibit further protein synthesis.
Instrument	Beckman Coulter Cytoflex S
Software	CytExpert 2.5
Cell population abundance	There was no sorting of cells. Maximum 60000 cell events were recorded with less than 20% of abortion rate.
Gating strategy	Events were gated by FSC area vs. SSC area to locate the cells and eliminate debris. Then the gated cells were further gated by SSC area and SSC height. This population was gated by FITC height value. The population having higher than 3000 FITC height value is considered as GFP ON, and that lower than 3000 considered as GFP OFF. For experiments quantifying mKate expression, ECD-H channel was used for gating. The population higher than 2000 ECD-H value is considered as mKate ON, and that lower than 2000 considered as mKate OFF. The details of Gating strategy is described in SI Fig. 14.

☒ Tick this box to confirm that a figure exemplifying the gating strategy is provided in the Supplementary Information.
